# Pyridylidene ligand facilitates gold-catalyzed oxidative C–H arylation of heterocycles

**DOI:** 10.3762/bjoc.11.295

**Published:** 2015-12-28

**Authors:** Kazuhiro Hata, Hideto Ito, Yasutomo Segawa, Kenichiro Itami

**Affiliations:** 1 Graduate School of Science, Nagoya University, Chikusa, Nagoya 464-8602, Japan; 2 JST, ERATO, Itami Molecular Nanocarbon Project, Nagoya University, Chikusa, Nagoya 464-8602, Japan; 3 Institute of Transformative Bio-Molecules (WPI-ITbM), Nagoya University, Chikusa, Nagoya 464-8602, Japan

**Keywords:** carbene ligand, C–H arylation, gold catalysis

## Abstract

Triaryl-2-pyridylidene effectively facilitates the gold-catalyzed oxidative C–H arylation of heteroarenes with arylsilanes as a unique electron-donating ligand on gold. The employment of the 2-pyridylidene ligand, which is one of the strongest electron-donating N-heterocyclic carbenes, resulted in the rate acceleration of the C–H arylation reaction of heterocycles over conventional ligands such as triphenylphosphine and a classical N-heterocyclic carbene. In situ observation and isolation of the 2-pyridylidene-gold(III) species, as well as a DFT study, indicated unusual stability of gold(III) species stabilized by strong electron donation from the 2-pyridylidene ligand. Thus, the gold(I)-to-gold(III) oxidation process is thought to be facilitated by the highly electron-donating 2-pyridylidene ligand.

## Introduction

Over the past decade, gold salts and complexes have emerged as unique catalysts for the transformation of alkynes, alkenes and allenes [1–30]. In most of the gold-catalyzed reactions, phosphines, N-heterocyclic carbenes, pyridines and salen ligands have been applied as ligands for controlling the stability of catalysts, and chemo-, regio- and enantioselectivities of the reactions [31–36]. Recent advances in the gold-catalyzed reactions are represented by oxidative coupling that is expected to proceed through a gold(I)/gold(III) catalytic cycle [37–81]. In particular, the elegant works of Lloyd-Jones and Russell on gold-catalyzed oxidative C–H arylation of simple arenes with arylsilanes have led the way to novel gold-catalyzed reactions that could not be achieved with other transition metals [68–69]. In these reactions, the oxidation of gold(I) to gold(III) is thought to be a key step in the catalytic cycle consisting of transmetalation with arylsilane, C–H activation and reductive elimination [69]. While gold(I) complexes bearing various ligands are used as gold(III) precursors, it remains unclear whether ligands can still coordinate to the gold center or not under such oxidative reaction conditions. For example, triphenylphosphine is easily oxidized to triphenylphosphine oxide by a hypervalent iodine reagent that has been used as an oxidant for gold-catalyzed C–H arylation [69]. Appropriate ligands that are tolerant to the oxidative conditions would offer numerous benefits such as high activity and stability of gold catalyst, thereby achieving otherwise-difficult oxidative transformations [37–40].

**Figure 1:**
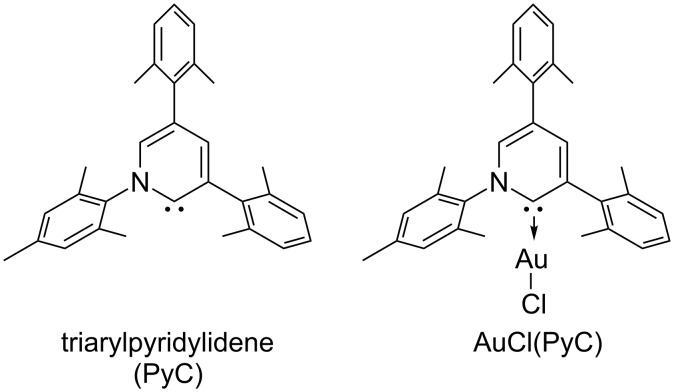
Triaryl-2-pyridylidene (PyC) and PyC-gold(I) complex (AuCl(PyC)).

Recently, we have introduced highly electron-donating triaryl-2-pyridylidene (PyC: pyridine-based carbene) [82–84] as a new type of nonclassical N-heterocyclic carbene [85–102]. We demonstrated that the PyC ligand is one of the strongest electron-donating carbene ligands to a gold(I) species (Figure 1) [83]. The AuCl(PyC) complex is very stable, even in air and moisture, and isolable by column chromatography on silica gel. Thus we envisioned that a gold complex with strongly electron-donating PyC would promote the gold(I)-to-gold(III) oxidation process, facilitating oxidative coupling reactions. Herein we report that the PyC ligand facilitates gold-catalyzed oxidative C–H arylation of hereroarenes that has been known to be very sluggish with typical ligand systems [68–72]. In this paper, the C–H arylation reactions of isoxazole, indole, and benzothiophene are presented. In addition, direct observation and isolation of PyC-gold(III) complexes are described.

## Results and Discussion

### Ligand effect of PyC in gold-catalyzed aromatic C–H arylation

In this study, we selected the gold-catalyzed oxidative C–H arylation of arenes with arylsilanes [68–69], reported by Lloyd-Jones and Russell, to test the ligand effect of PyC (Table 1). Likely due to the low stability of electron-rich heteroarene substrates toward oxidative conditions [103–107], their original conditions usually do not work well for these substrates. For example, when isoxazole (**1a**: 1 equiv) [108–111] was treated with 1-bromo-4-(trimethylsilyl)benzene (**2a**: 1 equiv) in chloroform/methanol solution at 65 °C in the presence of AuCl(PPh_3_) (5 mol %), iodosobenzoic acid (IBA: 1 equiv) and (+)-10-camphorsulfonic acid (CSA: 1 equiv), the corresponding C–H arylation product **3aa** was obtained in only 10% yield (Table 1, entry 1). Although the application of IPr, a conventional NHC ligand, to the reaction did not afford **3aa** at all (Table 1, entry 2), PyC promoted the reaction with higher yield of 4-arylisoxazole **3aa** under these conditions (30%, Table 1, entry 3). In the AuCl(PyC)-catalyzed reaction, **1a** was fully consumed, and 4,4’-dibromobiphenyl (**4a**) derived from the homocoupling of arylsilane **2a** was also detected. Furthermore, a significant amount of methyl 2-iodobenzoate (**5**) was generated through the esterification of a co-product (2-iodobenzoic acid) with methanol. We also tested other iodine(III) reagents such as PhI(OAc)_2_, PhI(OCOCF_3_)_2_ and PhI(OH)(OTs), but they all resulted in lower yields than IBA mainly due to the formation of diaryliodonium PhI(4-BrC_6_H_4_)^+^ produced by the reaction with arylsilane **2a** (Table 1, entries 4–6) [69]. Using *p*-toluenesulfonic acid (TsOH) instead of CSA was less effective (Table 1, entry 7). It was clearly seen that both CSA and methanol had a significant effect on the reaction progress (Table 1, entries 8 and 9). Nevertheless, the highest yield achieved by the use of AuCl(PyC) may be attributed to the highly electron-donating nature of the PyC ligand.

**Table 1:** Effect of ligand and oxidant in gold-catalyzed oxidative C–H arylation of isoxazole **1a**.^a^

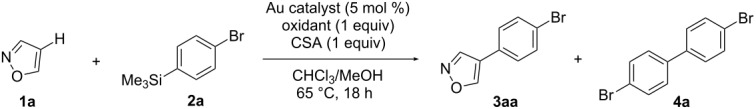				

Entry	Au catalyst	Oxidant	Yield [%]^b^	

			**3aa**	**4a**

1	AuCl(PPh_3_)	IBA	10	7
2	AuCl(IPr)	IBA	0	0
3	AuCl(PyC)	IBA	30	12
4	AuCl(PyC)	PhI(OAc)_2_	4	5
5	AuCl(PyC)	PhI(OCOCF_3_)_2_	3	4
6^c^	AuCl(PyC)	PhI(OH)(OTs)	9	5
7^d^	AuCl(PyC)	IBA	13	5
8^c^	AuCl(PyC)	IBA	0	9
9^e^	AuCl(PyC)	IBA	3	36

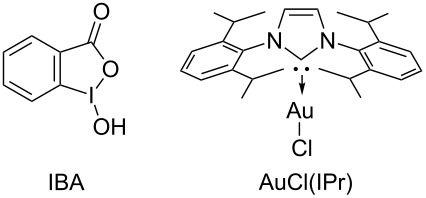
^a^Reaction conditions: **1a** (0.20 mmol), **2a** (0.20 mmol), Au catalyst (5 mol %), oxidant (0.20 mmol), (+)-10-camphorsulfonic acid (CSA, 0.20 mmol), CHCl_3_/MeOH (10:1, 1.1 mL), 65 °C. ^b^Determined by GC analysis with *n*-nonane as an internal standard. ^c^Without CSA. ^d^TsOH·H_2_O was used instead of CSA. ^e^CHCl_3_ (1.0 mL) was used as solvent.

### Oxidative C–H arylation of heteroarenes with arylsilanes catalyzed by AuCl(PyC)

Having discovered the positive effect of using PyC as a ligand, we further examined the C–H arylation of various heteroarenes with arylsilanes (Table 2). It should be noted that all of the examined heteroarenes were not successfully applied in the previous gold-catalyzed C–H arylation. The reactions of **1a** with halogenated aryltrimethylsilanes **2a** and **2b** afforded coupling products **3aa** and **3ab** in 14% and 15% isolated yields, respectively (Table 2, entries 1 and 2) [112]. 5-Methylisoxazole (**1b**) was arylated with bromo-, fluoro- and trifluoromethyl-substituted aryltrimethylsilanes, **2a**, **2b** and **2c**, respectively, to give the corresponding 4-aryl-5-methylisoxazoles, **3ba**, **3bb** and **3bc**, respectively, in higher efficiency as compared with **1a** (Table 2, entries 3–5). This may be due to the higher tolerability of **1b** than **1a** toward undesired decomposition [113]. The introduction of the 3,5-dibromophenyl group onto methylisoxazole **1b** resulted in lower yield of heterobiaryl **3bd** (Table 2, entry 6). In the reaction of 5-phenylisoxazole (**1c**), the selective arylation at the C4 position occurred without any arylation at the phenyl group (Table 2, entry 7). 3,5-Dimethylisoxazole (**1d**) showed low reactivity, likely due to the steric hindrance, but the reaction gave sterically congested heterobiaryl **3da** in 17% yield (Table 2, entry 8). In the case of the reaction of indole **1e**, 3-arylindole **3ea** was exclusively obtained in 44% yield (Table 2, entry 9). On the other hand, arylation of benzo[*b*]thiophene (**1f**) mainly afforded 2-arylbenzothiophene **3fa** along with a small amount of 3-arylbenzothiophene **3fa'** (Table 2, entry 10).

**Table 2:** AuCl(PyC)-catalyzed oxidative C–H arylation of heteroarenes with arylsilanes.^a^

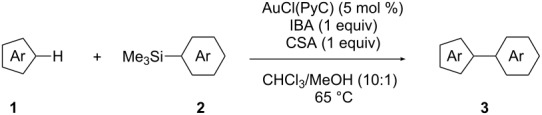				

Entry	**1**	**2**	**3**	Yield^b^

1	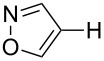 **1a**	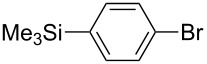 **2a**	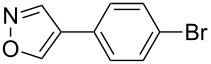 **3aa**	14%
2	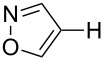 **1a**	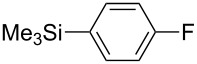 **2b**	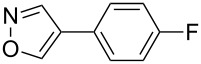 **3ab**	15%
3	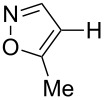 **1b**	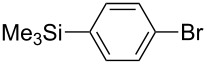 **2a**	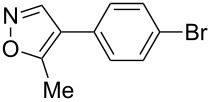 **3ba**	55%
4	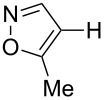 **1b**	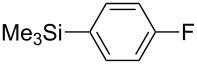 **2b**	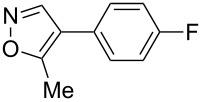 **3bb**	54%
5	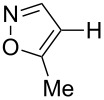 **1b**	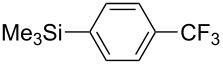 **2c**	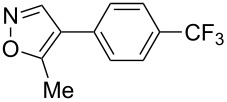 **3bc**	33%
6	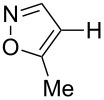 **1b**	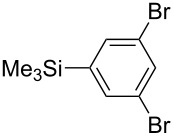 **2d**	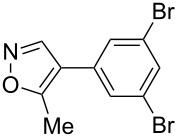 **3bd**	13%
7	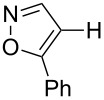 **1c**	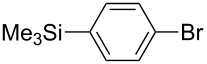 **2a**	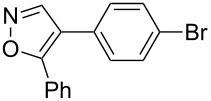 **3ca**	28%
8	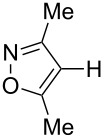 **1d**	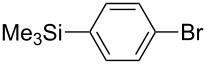 **2a**	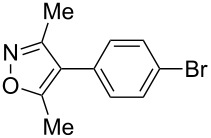 **3da**	17%
9	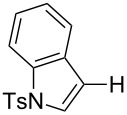 **1e**	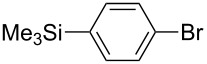 **2a**	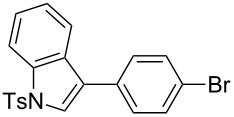 **3ea**	44%
10	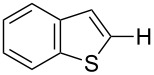 **1f**	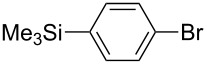 **2a**	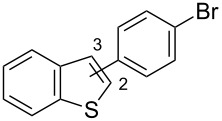 **3fa** (C2):**3fa’** (C3) = 83:17	22%

^a^Reaction conditions: **1** (0.20 mmol), **2** (0.20 mmol), AuCl(PyC) (5 mol %), IBA (0.20 mmol), CSA (0.20 mmol), CHCl_3_/MeOH (10:1, 1.1 mL), 65 °C, 18–48 h. ^b^Isolated yield.

### Reaction progress analysis

**Figure 2:**
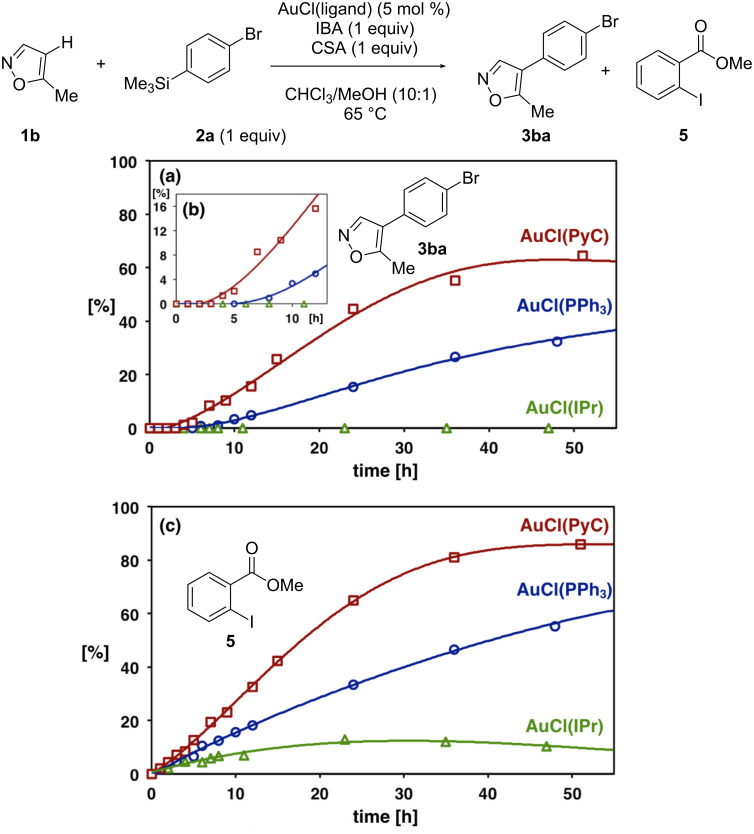
Yield–time profiles of 4-(4-bromophenyl)-5-methylisoxazole (**3ba**) and methyl 2-iodobenzoate (**5**) with AuCl(PyC), AuCl(PPh_3_) and AuCl(IPr). (a) Yield of **3ba**. (b) Magnified figure of (a). (c) Yield of **5**. All yields were determined by GC analysis with *n*-nonane as an internal standard.

To further unveil the ligand effect of PyC, time-production profiles of coupling product **3ba** were investigated for the reaction of **1b** and **2a** with AuCl(PyC), AuCl(PPh_3_) and AuCl(IPr). The yield of **3ba** was determined by GC analysis, whereas the consumption of IBA (oxidant) was estimated by the production of methyl 2-iodobenzoate (**5**). The reaction plots with AuCl(PyC), AuCl(PPh_3_) and AuCl(IPr) are depicted in Figure 2. Noteworthy observations are as follows: (i) the reaction with AuCl(PyC) was fastest among those with three catalysts (Figure 2a), (ii) the induction periods with regard to the formation of **3ba** were found in the reactions using AuCl(PyC) and AuCl(PPh_3_) (Figure 2a,b), and (iii) the oxidant consumption began at the reaction initiation for all catalysts (Figure 2c). In the reaction using AuCl(PyC), the coupling product **3ba** was generated after a shorter induction period of about 3 h and reached 60% yield after 50 h (Figure 2a). On the other hand, the reaction using AuCl(PPh_3_) began after a longer induction period (ca. 5 h), and the yield of **3ba** did not exceed the yield with AuCl(PyC) even after 100 h (see Supporting Information File 1 for details). No coupling product was produced with AuCl(IPr) although the consumption of about 10% of IBA was observed.

### Mechanistic considerations

**Scheme 1:**
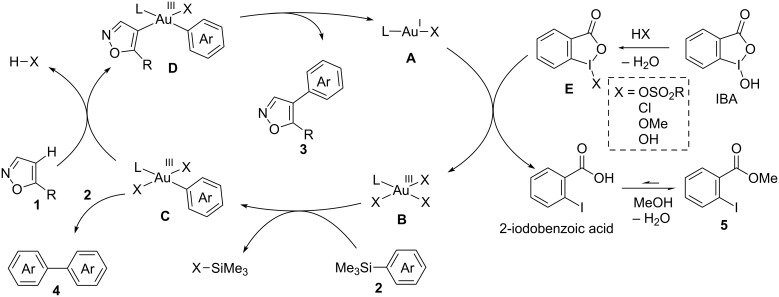
Plausible reaction mechanism of gold-catalyzed oxidative C–H arylation of heteroarenes with arylsilanes.

Based on the above results and the literature [68–75], we propose the reaction mechanism of the gold-catalyzed C–H arylation of heteroarenes with arylsilanes as shown in Scheme 1. A gold(I) complex **A** is first oxidized to gold(III) species **B** by the iodine(III) reagent **E** derived from IBA by the exchange of a hydroxy group with an existing acid such as CSA, HCl and MeOH. We independently confirmed that the esterification of 2-iodobenzoic acid takes place to give **5** under the reaction conditions; 2-iodobenzoic acid was smoothly converted to **5** in chloroform/methanol solution at 65 °C. Transmetalation of gold(III) complex **B** with arylsilane **2** affords monoarylated gold(III) intermediate **C**. The electrophilic metalation of heteroarene **1** with **C** with concurrent generation of an acid (HX) produces diarylated gold(III) species **D**. Finally, the reductive elimination from **D** releases the coupling product **3** along with the regeneration of gold(I) species **A**. The side reaction leading to the homocoupling product of arylsilane **4** likely occurs via over-transmetalation of monoarylated gold(III) species **C** with arylsilane **2** or disproportionation of **C** [67–81].

### Oxidation process of gold

In all reaction progress experiments with the three gold catalysts (Figure 2), the consumption of IBA (production of **5**) was observed to some extent even in the induction period. Taking the possible reaction mechanism into consideration, the oxidation of gold(I) to gold(III) by the oxidant may occur during the induction period. While it is unclear what is oxidized in these reactions, we hypothesize that the highly electron-donating PyC ligand facilitates the oxidation of gold(I) to gold(III). As triphenylphosphine is known to be easily oxidized to triphenylphosphine oxide under the current oxidative conditions, the ligand-free gold(III) species is thought to be an active species in the arylation reaction with AuCl(PPh_3_) [69]. While the IPr-gold(I) complex is known to undergo oxidation to an IPr-gold(III) species [114], its inactiveness in the current reaction indicates that the electron-donating capability is not high enough to facilitate this process.

### Direct observation and isolation of PyC-gold(III) complex

**Figure 3:**
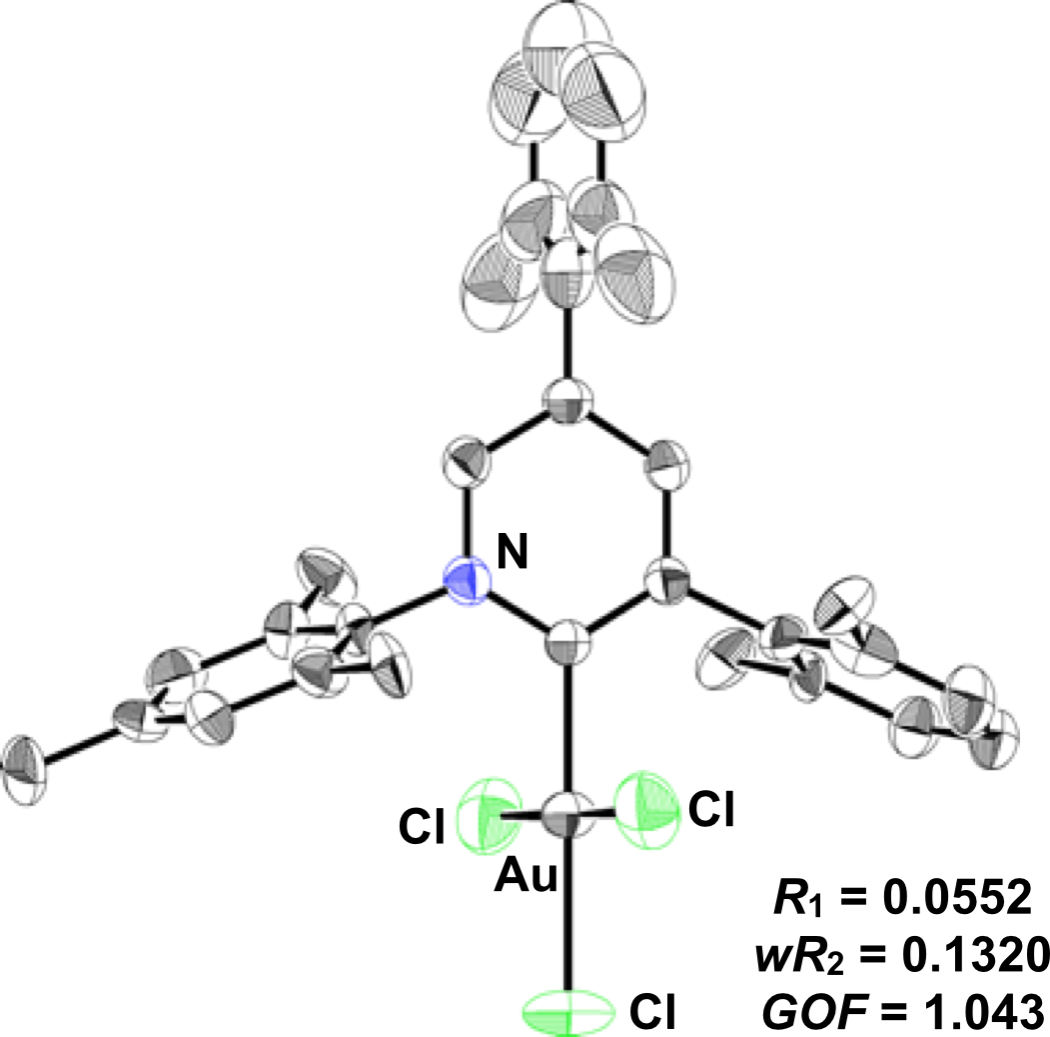
ORTEP drawing of AuCl_3_(PyC) with 50% probability. Hydrogen atoms and solvent are omitted for clarity.

To verify our hypothesis that PyC accelerates the gold(I)-to-gold(III) oxidation, we attempted the direct observation and the isolation of the PyC-gold(III) complex. First of all, the gold(III) complex AuCl_3_(PyC) was newly synthesized by treating AuCl(PyC) with PhICl_2_ (see Experimental section and Supporting Information File 1 for details) [114]. The X-ray crystallographic analysis was successfully accomplished with a colorless single crystal of AuCl_3_(PyC), which was recrystallized from nitrobenzene and pentane (Figure 3) [115]. The X-ray crystal structure shows that the four gold bonds are in a planar surface, and the pyridylidene face and the added two chlorine atoms are in vertical positions. The ligand arrangement is quite similar to a series of reported NHC–AuCl_3_ complexes [114].

**Scheme 2:**
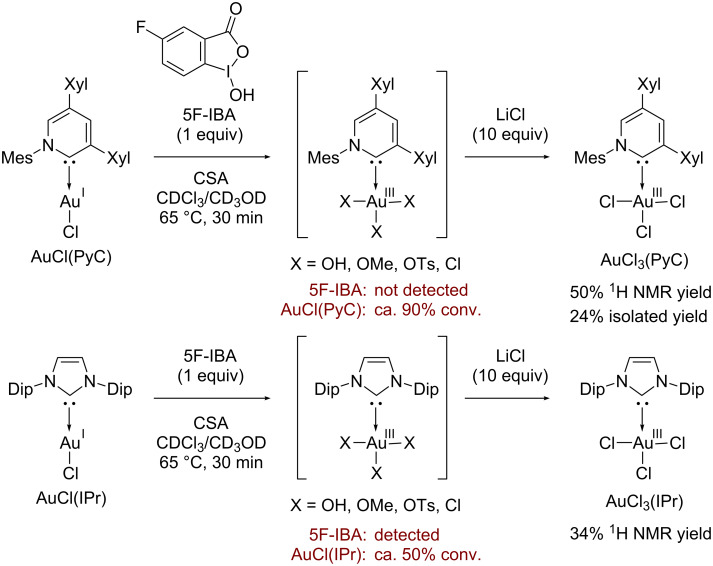
Direct observation and isolation of carbene-gold(III) complex. Mes = 2,4,6-Me_3_C_6_H_2_, Xyl = 2,6-Me_2_C_6_H_3_, Dip = 2,6-iPr_2_C_6_H_3_.

With the authentic AuCl_3_(PyC) in hand, we next carried out the direct observation of PyC-gold(III) species under the catalytic conditions. The treatment of AuCl(PyC) with 5-fluoroiodosobenzoic acid (5F-IBA) and CSA in CDCl_3_/CD_3_OD at 65 °C resulted in the full consumption of 5F-IBA within 30 min (monitored by ^19^F NMR). While the resulting mixture seemed to contain several PyC-gold(III) complexes, the formation of various gold(III) species bearing hydroxy, methoxy, sulfoxy and chloro groups made the analysis and isolation difficult. However, the subsequent addition of excess LiCl enabled us to detect the gold(III) species as AuCl_3_(PyC) by ^1^H and ^13^C NMR analyses. The ^1^H NMR analysis revealed that about 90% of AuCl(PyC) was consumed and AuCl_3_(PyC) was produced in 50% NMR yield. Fortunately, the isolation from the messy crude mixtures was accomplished to give AuCl_3_(PyC) in 24% isolated yield. We also conducted the same experiment with the AuCl(IPr) complex. From the ^19^F and ^1^H NMR analyses, approximately half of AuCl(IPr) and oxidant 5F-IBA remained unreacted after heating for 30 min, and AuCl_3_(IPr) was observed only in 34% ^1^H NMR yield [114] (Scheme 2).

These observations on gold(III) species support our hypothesis that the highly electron-donating PyC ligand strongly coordinates to a gold center and promotes the gold(I)-to-gold(III) oxidation by stabilizing a gold(III) species without dissociation. An IPr-gold(III) complex is known to be stable, but the lower electron-donation ability of IPr than that of PyC seems to result in the inefficient oxidation of AuCl(IPr). DFT calculations on the oxidation process of the AuCl(ligand) to AuCl_3_(ligand) also clarified the advantage of the PyC ligand over IPr by 3.6 kcal mol^–1^ (see Supporting Information File 1 for details). While it still remains unclear how the PyC ligand affects the transmetalation, C–H metalation and reductive elimination steps, we believe that the strongly electron-donating PyC not only facilitates gold(I)-to-gold(III) oxidation in catalysis but also prolongs the catalyst lifetime by preventing the ligand dissociation and formation of inactive gold nanoparticles.

## Conclusion

In summary, we have developed the oxidative C–H arylation of heteroarenes with arylsilanes catalyzed by PyC-gold complex and revealed the advantageous features of using the PyC ligand. From the reaction progress, experiments and stoichiometric oxidation of gold(I) complexes, we conclude that the highly electron-donating PyC ligand promotes the gold(I)-to-gold(III) oxidation and stabilizes the gold(III) species, thereby facilitating the oxidative coupling reactions.

## Experimental

**Preparation of triarylpyridylidene-gold(I) chloride [AuCl(PyC)]:** A 10 mL Schlenk tube containing a stir bar was dried under vacuum and filled with N_2_ after cooling to room temperature. Ag_2_O (232 mg, 1.0 mmol) and NBu_4_Cl·H_2_O (1.39 g, 5.0 mmol) were added to the solution of 3,5-bis(2,6-dimethylphenyl)-1-mesitylpyridin-1-ium triflate (730 mg, 1.0 mmol) in 1,2-dichloroethane (5.0 mL). The mixture was stirred at room temperature for 2 h, and AuCl(SMe_2_) (11.5 mg, 0.10 mmol) was then added to the reaction mixture. The reaction mixture was further stirred overnight, and the addition of CHCl_3_ (50 mL) to the mixture gave a white precipitate. The suspension was filtered off and the filtrate was concentrated under reduced pressure. The crude product was purified by column chromatography on silica gel (eluents: MeOH/CHCl_3_ 1:20) and recrystallized from CHCl_3_/toluene at room temperature to give a pure AuCl(PyC)/toluene complex (286 mg, 40%) as a pale yellow crystal. The addition of CHCl_3_ and the concentration in vacuum yielded a pure AuCl(PyC) complex without toluene as white powder. The characterization data for AuCl(PyC) corresponded to the reported values [83].

**General procedure for AuCl(PyC)-catalyzed oxidative C–H arylation of heteroarenes with arylsilanes:** AuCl(PyC) (6.4 mg, 0.010 μmol, 5.0 mol %), heteroarene **1** (0.20 mmol), and aryltrimethylsilane **2** (0.20 mmol), 2-iodosobenzoic acid (IBA, 53 mg, 0.20 mmol), 10-camphorsulfonic acid (CSA) (47 mg, 0.20 mmol) and a stir bar were placed in a screw test tube, and dry CHCl_3_/MeOH (1.0 mL/0.10 mL) was added under N_2_ atmosphere. The tube was sealed with a cap equipped with a Teflon^®^-coated silicon rubber septum, and the mixture was stirred at 65 °C for 18–48 h. The reaction was quenched by addition of excess saturated aqueous NaHCO_3_, the aqueous layer was extracted with CH_2_Cl_2_, and the combined organic layers were dried over Na_2_SO_4_, filtered, and concentrated under reduced pressure. The residue was purified by flash chromatography on silica gel to afford the coupling product **3** (Table 2).

**Oxidation of AuCl(PyC):** The oxidation of AuCl(PyC) was performed according to the literature [16]. PhICl_2_ (54.8 mg, 0.20 mmol) was added into a solution of AuCl(PyC) (128 mg, 0.20 mmol) in CH_2_Cl_2_ (2.0 mL) under N_2_ atmosphere. After stirring at room temperature for 19 h, the reaction mixture was filtered through a pad of Celite^®^. The filtrate was poured into hexane and the resulting precipitate was collected by filtration to obtain pure AuCl_3_(PyC) as a white solid (140 mg, 99%). The colorless single crystal used for X-ray diffraction analysis was obtained by recrystallization from nitrobenzene and pentane. ^1^H NMR (CDCl_3_, 600 MHz) δ 8.17 (d, *J* = 2.1 Hz, 1H), 7.90 (d, *J* = 2.1 Hz, 1H), 7.29 (td, *J* = 7.6, 2.7 Hz, 2H), 7.19 (d, *J* = 7.6 Hz, 2H), 7.16 (d, *J* = 7.6 Hz, 2H), 7.07 (s, 2H), 2.36 (s, 3H), 2.28 (s, 12H), 2.15 (s, 6H); ^13^C NMR (CDCl_3_, 150 MHz) δ 162.5 (CH), 149.6 (4°), 146.6 (CH), 144.7 (CH), 141.9 (4°), 141.5 (4°), 138.5 (4°), 136.4 (4°), 135.6 (4°), 135.6 (4°), 133.2 (4°), 132.2 (4°), 130.4 (CH), 129.9 (CH), 129.7 (CH), 128.3 (4°), 128.3 (CH), 22.1 (CH_3_), 21.1 (CH_3_), 20.9 (CH_3_), 19.3 (CH_3_); HRMS (ESI+) *m*/*z*: [M − Cl + MeOH]^+^ calcd for C_31_H_35_AuCl_2_NO, 704.1756; found, 704.1722.

**In situ observation and isolation of AuCl_3_(PyC):** AuCl(PyC) (12.8 mg, 0.020 mmol), 5-fluoroiodosobenzoic acid (5F-IBA, 5.6 mg, 0.020 mmol) and CSA (4.6 mg, 0.020 mmol) were placed in an NMR tube, and CDCl_3_/CD_3_OD (10:1, 0.60 mL) was added under N_2_ atmosphere. The tube was sealed with a cap equipped with a Teflon^®^-coated silicon rubber septum and heated at 65 °C for 30 min. After cooling to room temperature, LiCl (8.4 mg, 0.20 mmol) was added. 1,1,2,2-Tetrachloroethane was added as an internal standard and an NMR yield of AuCl_3_(PyC) was estimated by ^1^H NMR spectroscopy. The solvent was removed in vacuum, and the residue was dissolved in EtOAc. The organic layer was washed with saturated aqueous NaHCO_3_ and brine, dried over Na_2_SO_4_, filtered, and concentrated in vacuum to afford the crude mixture. The crude mixture was further washed with Et_2_O to give pure AuCl_3_(PyC) as a white powder (3.4 mg, 24%, Scheme 2).

## Supporting Information

File 1 Experimental procedures, spectra of new compounds, CIF data, and details of the computational study.
