# Supplementary material for: Pyridylidene ligand facilitates gold-catalyzed oxidative C–H arylation of heterocycles
Source: Beilstein J Org Chem. 2015 Dec 28;11:2737–46. doi: 10.3762/bjoc.11.295 (PMC4734412; doi:10.3762/bjoc.11.295)

Supporting Information

for

Pyridylidene ligand facilitates gold-catalyzed oxidative C–H arylation of heterocycles

Kazuhiro Hata[1], Hideto Ito[1], Yasutomo Segawa*[1,2] and Kenichiro Itami*[1,2,3]

Address: 1Graduate School of Science, Nagoya University, Chikusa, Nagoya 464-8602, Japan, 2JST, ERATO, Itami Molecular Nanocarbon Project, Nagoya University, Chikusa, Nagoya 464-8602, Japan and 3Institute of Transformative Bio-Molecules (WPI-ITbM), Nagoya University, Chikusa, Nagoya 464-8602, Japan

Email: Yasutomo Segawa - ysegawa@nagoya-u.jp; Kenichiro Itami - itami@chem.nagoya-u.ac.jp

* Corresponding author

Experimental procedures, spectra of new compounds, CIF data, and details of the computational study

Table of Contents

General	S2
Preparation of (triarylpyridylidene)gold chloride	S3
Oxidation of (triarylpyridylidene)gold chloride	S3
In situ observation and isolation of (triarylpyridylidene)gold trichloride	S4
General procedure for triarylpyridylidene-gold-catalyzed oxidative C–H arylation of heteroarenes with arylsilanes	S4
General procedure for GC analysis of reaction profiles of each reaction component	S5
Characterization of new compounds	S6
X-ray crystallography	S11
Computational study	S13
References	S16
^{1}H and ^{13}C NMR spectra	S17

General

Unless otherwise noted, all materials including dry solvents were purchased from commercial suppliers and used without further purification. All reactions were performed using standard vacuum-line, Schlenk techniques, and screw cap tube. Work-up and purification procedures were carried out with reagent-grade solvents under air.

Analytical thin-layer chromatography (TLC) was carried out on Merck silica gel 60 F_{254} plates; detection by UV or dipping into a solution of $Ce(SO_4)_2\cdot H_2O$ (10 g), phosphomolybdic acid hydrate (25 g), conc. H_2SO_4 (60 mL) and H_2O (0.94 L) or $NaHCO_3$ (5.0 g), $KMnO_4$ (1.5 g) and H_2O (0.20 L) followed by heating. Flash chromatography (FC) was carried out on Merck or KANTO silica gel 60 (40–100 μm) with an air pressure of about 1.1–1.5 bar. Preparative thin-layer chromatography (PTLC) was performed using Wako-gel® B5-F silica coated plates (0.75 mm) prepared in our laboratory. High-resolution mass spectra (HRMS) were measured on a Thermo Fisher Exactive Plus spectrometer (ESI) and (APCI). Nuclear magnetic resonance (NMR) spectra were recorded on *JEOL* JNM-ECA-600 (^{1}H: 600 MHz, ^{13}C: 150 MHz). Chemical shifts for ^{1}H NMR are expressed in parts per million (ppm) relative to $CHCl_3$ (δ 7.26 ppm). Chemical shifts for ^{13}C NMR are expressed in ppm relative to $CHCl_3$ (δ 77.0 ppm). Data are reported as follows: chemical shift, multiplicity (s = singlet, d = doublet, dd = doublet of doublets, dt = doublet of triplets, t = triplet, m = multiplet), coupling constant (Hz), and integration.

Preparation of (triarylpyridylidene)gold chloride [AuCl(PyC)]

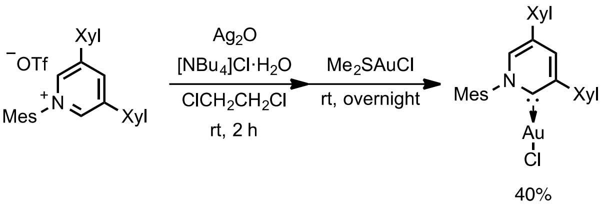

Silver(I) oxide (232 mg, 1.0 mmol) was added into a reaction vessel filled with an 1,2-dichloroethane (5.0 mL) solution of 3,5-bis(2,6-dimethylphenyl)-1-mesityl-pyridinium triflate·toluene[S1] (648 mg, 1.0 mmol) and tetrabutylammonium chloride hydrate (1.4 g, 5.0 mmol) under N_2 atmosphere. The reaction mixture was stirred at room temperature in dark until the silver(I) oxide powder disappeared (1–2 h) before chloro(dimethylsulfide)gold(I) was added. Further stirring overnight afforded a yellow solution and a black solid. Addition of chloroform (10 mL) gave an insoluble white precipitation, and these solids were removed by Celite® filtration and the filtrate was concentrated under reduced pressure. The crude oily mixture was purified by column chromatography on silica gel with chloroform/methanol (20:1) as the eluents and the fractions were concentrated under reduced pressure. Reprecipitation from chloroform/toluene gave colorless microcrystals. Further purification was conducted by recrystallization from chloroform/toluene to produce AuCl(PyC)·toluene as white crystals (281 mg, 0.40 mmol, 40% yield). Toluene in the crystal was removed by evaporation of chloroform solution. The characterization data for compound AuCl(PyC) corresponded to the reported values.[S1]

Oxidation of (triarylpyridylidene)gold chloride

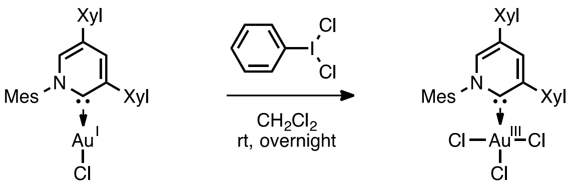

Oxidation of AuCl(PyC) was performed according to the literature.[S2] dichloro(phenyl)-λ^3-iodane (55 mg, 0.20 mmol) was added into the solution of AuCl(PyC) (128 mg, 0.200 mmol) in dichloromethane (2.0 mL) under N_2 atmosphere. The reaction mixture was stirred at room temperature for 19 h and was filtered through a pad of Celite®. The filtrate was poured into hexane and the resulting precipitate was collected by filtration to obtain pure $AuCl_3$(PyC) as a white solid (140 mg, 99%). A colorless single crystal for X-ray diffraction analysis was obtained by recrystallization from nitrobenzene and pentane.

In situ observation and isolation of (triarylpyridylidene)gold trichloride

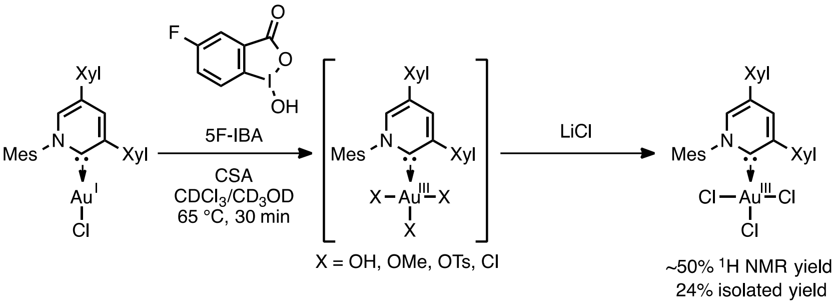

AuCl(PyC) (13 mg, 20 μmol), 5-fluoro-2-iodosobenzoic acids (5F-IBA) (5.6 mg, 20 μmol) and (+)-10-camphorsulfonic acid (CSA) (4.6 mg, 20 μmol) were placed in a screw NMR tube, and $CDCl_3/CD_3OD$ (10:1, 0.60 mL) was added under N_2 atmosphere. The tube was sealed with a cap equipped with a Teflon®-coated silicon rubber septum, and heated at 65 °C for 30 min. After cooling to room temperature, lithium chloride (8.4 mg, 0.20 mmol) was added. Then 1,1,2,2-tetrachloroethane (an internal standard) was added and the NMR yield of $AuCl_3$(PyC) was estimated by ^{1}H NMR. The solvent was removed in vacuo, and the residue was dissolved in ethyl acetate, the organic layer was washed with saturated $NaHCO_3$ aq, and brine, dried over Na_2SO_4, filtrated and concentrated in vacuo to afford a crude mixture. The crude mixture was further washed with Et_2O to give pure $AuCl_3$(PyC) as white powder (3.4 mg, 24%).

General procedure for triarylpyridylidene-gold-catalyzed oxidative C–H arylation of heteroarenes with arylsilanes

Triarylpyridylidene-gold complex AuCl(PyC) (6.4 mg, 10 μmol, 5 mol %), heteroarene (0.20 mmol), and aryl(trimethyl)silane (0.20 mmol), 2-iodosobenzoic acids (IBA) (53 mg, 0.20 mmol), (+)-10-camphorsulfonic acid (CSA) (47 mg, 0.20 mmol) and a stirring bar were placed in a screw test tube, and dry chloroform/methanol (1.0 mL/0.10 mL) was added under N_2 atmosphere. The tube was sealed with a cap equipped with a Teflon®-coated silicon rubber septum, and the mixture was stirred at 65 °C for 18–48 h. The reaction was quenched by addition of excess saturated $NaHCO_3$ aq, the aqueous layer was extracted with dichloromethane and the combined organic layers were dried over Na_2SO_4, filtrated, and concentrated under reduced pressure. The residue was purified by flash chromatography (FC) to afford the coupling product.

General procedure for GC analysis of reaction profiles of each reaction component

A gold complex [AuCl(ligand)] (25 μmol, 5 mol %), 5-methylisoxazole (**1b**) (42 mg, 0.50 mmol), (4-bromophenyl)(trimethyl)silane (**2a**) (115 mg, 0.50 mmol), 2-iodosobenzoic acids (IBA) (132 mg, 0.50 mmol), (+)-10-camphorsulfonic acid (CSA) (116 mg, 0.50 mmol), the internal standard (nonane, 50 μL) and a stirring bar were placed in a Schlenk tube, and dry chloroform/methanol (5.0 mL/0.50 mL) solution was added under N_2 atmosphere. The tube was sealed with a glass stopper, and the mixture was stirred at 65 °C. A tiny portion of reaction mixture was taken with a capillary under N_2 atmosphere, and its diluted solution was analyzed by GC.

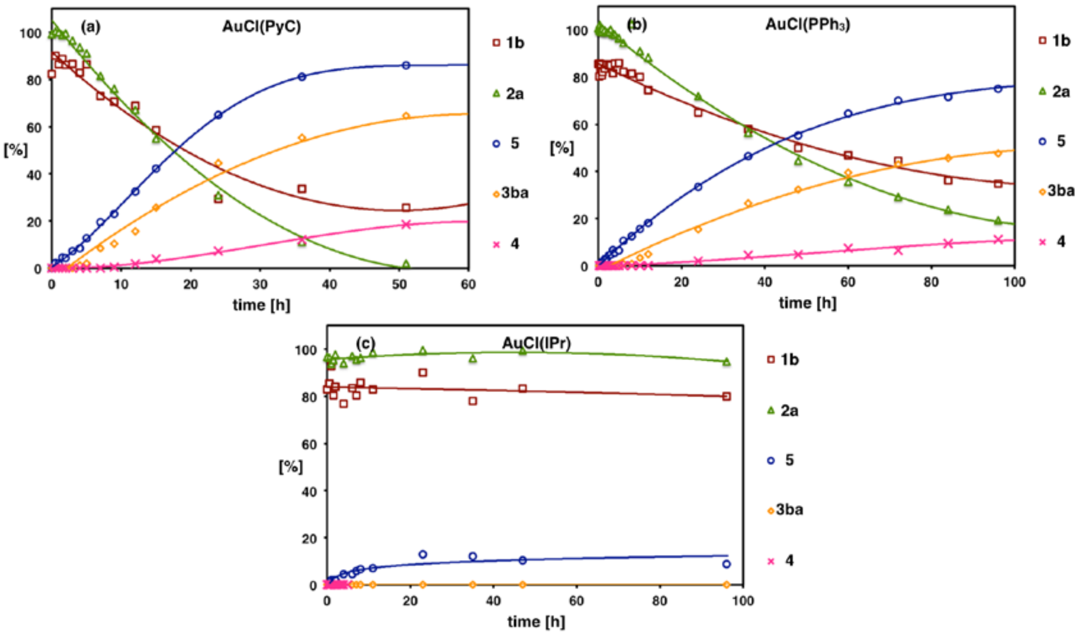

Figure S: Time-yield/consumption profiles of 5-methylisozxazole (**1b**), (4-bromophenyl)(trimethyl)silane (**2a**), 4-(4-bromophenyl)-5-methylisoxazole (**3ba**) methyl 2-iodobenzoate (**5**) and 4,4'-dibromobiphenyl (**4**) with (a) AuCl(PyC), (b) AuCl(PPh_3) and (c) AuCl(IPr). All yields were determined by GC analysis with *n*-nonane as an internal standard

Characterization of new compounds

[3,5-Bis(2,6-dimethylphenyl)-1-mesityl-pyridylidene]gold trichloride [$AuCl_3$(PyC)]

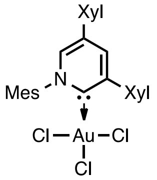

^{1}H NMR ($CDCl_3$, 600 MHz) δ 8.17 (d, J = 2.1 Hz, 1H), 7.90 (d, J = 2.1 Hz, 1H), 7.29 (td, J = 7.6, 2.7 Hz, 2H), 7.19 (d, J = 7.6 Hz, 2H), 7.16 (d, J = 7.6 Hz, 2H), 7.07 (s, 2H), 2.36 (s, 3H), 2.28 (s, 12H), 2.15 (s, 6H); ^{13}C NMR ($CDCl_3$, 150 MHz) δ 162.5 (CH), 149.6 (4°), 146.6 (CH), 144.7 (CH), 141.9 (4°), 141.5 (4°), 138.5 (4°), 136.4 (4°), 135.62 (4°), 135.58 (4°), 133.2 (4°), 132.2 (4°), 130.4 (CH), 129.9 (CH), 129.7 (CH), 128.32 (4°), 128.29 (CH), 22.1 (CH_3), 21.1 (CH_3), 20.9 (CH_3), 19.3 (CH_3); HRMS (ESI+) calcd for $C_{31}H_{35}AuCl_2NO$ [M–Cl+MeOH]$^+$: *m/z* 704.1756, found: 704.1722.

4-(4-Bromophenyl)isoxazole (**3aa**)

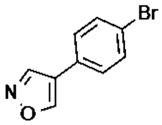

3aa was prepared according to the general procedure, employing isoxazole (**1a**) (14 mg, 0.20 mmol) and (4-bromophenyl)(trimethyl)silane (**2a**) (46 mg, 0.20 mmol) for 18 h. FC (hexane/ethyl acetate = 20:1 to 10:1) gave **3aa** as a pale yellow solid (6.1 mg, 0.027 mmol, 14%).

^{1}H NMR ($CDCl_3$, 600 MHz) δ 8.67 (s, 1H), 8.53 (s, 1H), 7.56–7.53 (m, 2H), 7.36–7.33 (m, 2H); ^{13}C NMR ($CDCl_3$, 150 MHz) δ 153.5 (CH), 147.7 (CH), 132.3 (CH), 127.9 (CH), 127.5 (4°), 122.0 (4°), 120.4 (4°); HRMS (ESI+) calcd for C_9H_7BrNO [M+H]$^+$: *m/z* 223.9706, found: *m/z* 223.9695.

4-(4-Fluorophenyl)isoxazole (**3ab**)

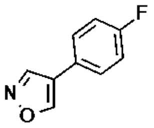

3ab was prepared according to the general procedure, employing isoxazole (**1a**) (14 mg, 0.20 mmol) and (4-fluorophenyl)(trimethyl)silane (**2b**) (37 mg, 0.20 mmol) for 24 h. FC (pentane/diethylether = 10:1) gave **3ab** as a pale yellow solid (4.9 mg, 0.030 mmol, 15%).

^{1}H NMR ($CDCl_3$, 600 MHz) δ 8.64 (s, 1H), 8.52 (s, 1H), 7.46–7.43 (m, 2H), 7.14–7.10 (m, 2H); ^{13}C NMR ($CDCl_3$, 150 MHz) δ 162.5 (4°, J = 247 Hz), 153.2 (CH), 147.9 (CH), 128.2 (CH, J = 8.6 Hz), 124.7 (4°, J = 2.9 Hz), 120.5 (4°), 116.3 (CH, J = 21 Hz); HRMS (ESI+) calcd for C_9H_7FNO [M+H]$^+$: *m/z* 164.0506, found: *m/z* 164.0500.

4-(4-Bromophenyl)-5-methylisoxazole (**3ba**)

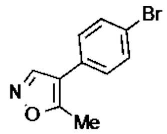

3ba was prepared according to the general procedure, employing 5-methylisoxazole (**1b**) (17 mg, 0.20 mmol) and (4-bromophenyl)(trimethyl)silane (**2a**) (46 mg, 0.20 mmol) for 24 h. FC (pentane/diethylether = 10:1) gave **3ba** as a pale yellow oil (26 mg, 0.11 mmol, 55%).

^{1}H NMR ($CDCl_3$, 600 MHz) [illegible]–7.23 (m, 2H), 2.55 (s, 3H); ^{13}C NMR ($CDCl_3$, 150 MHz) δ 164.5 (4°), 149.8 (CH), 132.1 (CH), 129.0 (4°), 128.9 (CH), 121.4 (4°), 115.5 (4°), 11.8 (CH_3); HRMS (ESI+) calcd for $C_{10}H_9BrNO$ $[M+H]^+$: *m/z* 237.9862, found: *m/z* 237.9855.

4-(4-Fluorophenyl)-5-methylisoxazole (**3bb**)

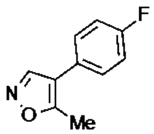

3bb was prepared according to the general procedure, employing 5-methylisoxazole (**1b**) (17 mg, 0.20 mmol) and (4-fluorophenyl)(trimethyl)silane (**2b**) (37 mg, 0.20 mmol) for 24 h. FC (pentane/diethylether = 10:1) gave **3bb** as a yellow oil (19 mg, 0.11 mmol, 54%).

^{1}H NMR ($CDCl_3$, 600 MHz) δ 8.31 (s, 1H), 7.35–7.31 (m, 2H), 7.15–7.10 (m, 2H), 2.54 (s, 3H); ^{13}C NMR ($CDCl_3$, 150 MHz) δ 164.2 (4°), 162.1 (4°, *J* = 247 Hz), 150.0 (CH), 129.1 (CH, *J* = 8.6 Hz), 126.1 (4°, *J* = 2.9 Hz), 116.0 (CH, *J* = 22 Hz), 115.6 (4°), 11.6 (CH_3); HRMS (ESI+) calcd for $C_{10}H_9FNO$ $[M+H]^+$: *m/z* 178.0663, found: *m/z* 178.0658.

4-(4-Trifluoromethylphenyl)-5-methylisoxazole (**3bc**)

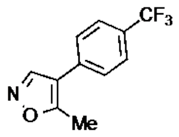

3bc was prepared according to the general procedure, employing 5-methylisoxazole (**1b**) (17 mg, 0.20 mmol) and (4-trifuoromethylphenyl)(trimethyl)silane (**2c**) (44 mg, 0.20 mmol) for 48 h. FC (hexane/ethyl acetate = 50:1 to 10:1) gave **3bc** as a colorless oil (15 mg, 0.066 mmol, 33%).

^{1}H NMR ($CDCl_3$, 600 MHz) δ 8.39 (s, 1H), 7.70 (d, J = 7.2 Hz, 2H), 7.50 (d, J = 8.4 Hz, 2H), 2.60 (s, 3H); ^{13}C NMR ($CDCl_3$, 150 MHz) δ 165.2 (4°), 149.8 (CH), 133.8 (4°), 129.6 (4°, J = 33 Hz), 127.6 (CH), 126.0 (CH, J = 4.4 Hz), 124.0 (4°, J = 270 Hz), 115.4 (4°), 11.9 (CH_3); HRMS (ESI+) calcd for $C_{11}H_9F_3NO$ $[M+H]^+$: m/z 228.0631, found: m/z 228.0622.

4-(3,5-Dibromo)-5-methylisoxazole (**3bd**)

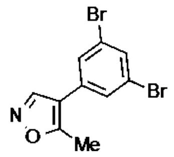

3bd was prepared according to the general procedure, employing 5-methylisoxazole (**1b**) (17 mg, 0.20 mmol) and (3,5-dibromophenyl)(trimethyl)silane (**2d**) (62 mg, 0.20 mmol) for 48 h. FC (hexane/ethylacetate = 50:1 to 10:1) gave **3bd** as a white solid (8.3 mg, 0.026 mmol, 13%).

^{1}H NMR ($CDCl_3$, 600 MHz) δ 8.33 (s, 1H), 7.64 (t, J = 1.8 Hz, 1H), 7.45 (d, J = 2.4 Hz, 2H), 2.58 (s, 3H); ^{13}C NMR ($CDCl_3$, 150 MHz) δ 165.3 (4°), 149.6 (CH), 133.6 (4°), 133.0 (CH), 129.1 (CH), 123.5 (4°), 114.2 (4°), 11.9 (CH_3); HRMS (ESI+) calcd for $C_{10}H_8Br_2NO$ $[M+H]^+$: m/z 317.8947, found: m/z 317.8935.

4-(4-Bromophenyl)-5-phenylisoxazole (**3ca**)

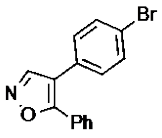

3ca was prepared according to the general procedure, employing 5-phenylisoxazole (**1c**) (29 mg, 0.20 mmol) and (4-bromophenyl)(trimethyl)silane (**2a**) (46 mg, 0.20 mmol) for 48 h. FC (hexane/ethyl acetate = 50:1 to 10:1) gave **3ca** as a white solid (17 mg, 0.057 mmol, 28%).

^{1}H NMR ($CDCl_3$, 600 MHz) δ 8.34 (s, 1H), 7.61 (dd, J = 8.1, 1.5 Hz, 2H), 7.54–7.51 (m, 2H), 7.45–7.38 (m, 3H), 7.27–7.24 (m, 2H); ^{13}C NMR ($CDCl_3$, 150 MHz) δ 164.3 (4°), 151.5 (CH), 132.2 (CH), 130.3 (CH), 130.2 (CH), 129.0 (4°), 128.9 (CH), 127.3 (4°), 127.3 (CH), 122.2 (4°), 115.1 (4°); HRMS (ESI+) calcd for $C_{15}H_{11}BrNO$ $[M+H]^+$: m/z 300.0019, found: m/z 300.0004.

4-(4-Bromophenyl)-3,5-dimethylisoxazole (**3da**)

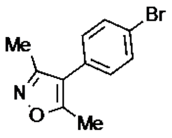

3da was prepared according to the general procedure, employing 3,5-dimethylisoxazole (**1d**) (19 mg, 0.20 mmol) and (4-bromophenyl)(trimethyl)silane (**2a**) (46 mg, 0.20 mmol) for 48 h. FC (pentane/dimethylether = 10:1) gave **3da** as a yellow solid (8.6 mg, 0.034 mmol, 17%).

^{1}H NMR ($CDCl_3$, 600 MHz) δ 7.57 (d, *J* = 8.4 Hz, 2H), 7.13 (d, *J* = 8.4 Hz, 2H), 2.39 (s, 3H), 2.25 (s, 3H); ^{13}C NMR ($CDCl_3$, 150 MHz) δ 165.3 (4°), 158.4 (4°), 132.0 (CH), 130.7 (CH), 129.4 (4°), 121.7 (4°), 115.7 (4°), 11.5 (CH_3) 10.7 (CH_3); HRMS (ESI+) calcd for $C_{11}H_{11}BrNO$ $[M+H]^+$: *m/z* 252.0019, found: *m/z* 252.0009.

3-(4-Bromophenyl)-*N*-tosylindole (**3ea**)

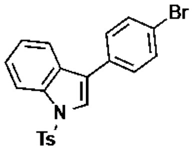

3ea was prepared according to the general procedure, employing *N*-*p*-toluenesulfonylindole (**1e**) (54 mg, 0.20 mmol) and (4-bromophenyl)(trimethyl)silane (**2a**) (46 mg, 0.20 mmol) for 48 h. FC (pentane/dimethylether = 10:1) gave **3ea** as a white solid (38 mg, 0.088 mmol, 44%).

^{1}H NMR ($CDCl_3$, 600 MHz) δ 8.07 (d, *J* = 8.4 Hz, 1H), 7.82 (d, *J* = 8.4 Hz, 2H), 7.72 (d, *J* = 8.4 Hz, 1H), 7.70 (s, 1H), 7.59 (d, *J* = 8.4 Hz, 2H), 7.47 (d, *J* = 8.4 Hz, 2H), 7.38 (t, *J* = 8.1 Hz, 1H), 7.30 (t, *J* = 7.5 Hz, 1H), 7.24 (d, *J* = 8.4 Hz, 2H), 2.34 (s, 3H); ^{13}C NMR ($CDCl_3$, 150 MHz) δ 145.1 (4°) 135.4 (4°), 135.1 (4°) 132.0 (CH), 130.0 (CH), 129.4 (CH), 128.9 (4°), 126.9 (CH), 125.0 (CH), 123.7 (CH), 123.0 (CH), 122.7 (4°), 121.4 (4°), 120.1 (CH), 113.9 (CH), 21.6 (CH_3), one 4° carbon peak should be overlapped; HRMS (ESI+) calcd for $C_{21}H_{17}BrNO_2S$ $[M+H]^+$: *m/z* 426.0158, found: *m/z* 426.0139.

2-(4-Bromophenyl)-benzo[*b*]thiophene (**3fa**) and 3-(4-bromophenyl)-benzo[*b*]thiophene (**3fa’**)

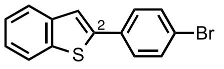

3fa

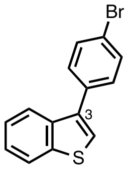

3fa'

3fa was prepared according to the general procedure, employing benzo[*b*]thiophene (**1f**) (27 mg, 0.20 mmol) and (4-bromophenyl)(trimethyl)silane (**2a**) (46 mg, 0.20 mmol) for 48 h. FC (pentane) gave **3fa** as mixture of **3fa'** (a white solid, 13 mg, 0.044 mmol, 22%, **3fa**:**3fa’** = 83:17). The characterization data for compound **3fa'** corresponded to the reported values.[S3]

^{1}H NMR of **3fa** ($CDCl_3$, 600 MHz) δ 7.83 (d, *J* = 8.4 Hz, 1H), 7.78 (d, *J* = 7.2 Hz, 1H), 7.59–7.53 (m, 5H), 7.38–7.32 (m, 2H); ^{13}C NMR of **3fa** ($CDCl_3$, 150 MHz) δ 142.9 (4°), 140.6 (4°), 139.5 (4°), 133.3 (4°), 132.1 (CH), 127.9 (CH), 124.7 (CH), 124.6 (CH), 123.7 (CH), 122.3 (CH), 122.2 (4°), 119.9 (CH); HRMS (APCI+) calcd for $C_{14}H_9BrS$ $[M]^+$: *m/z* 287.9603, found: *m/z* 287.9596.

X-ray crystallography

Details of the crystal data and a summary of the intensity data collection parameters for $AuCl_3$(PyC) are listed in Table S1. In each case, a suitable crystal was mounted with mineral oil on a glass fiber and transferred to the goniometer of a Rigaku Saturn CCD diffractometer. Graphite-monochromated Mo Kα radiation (λ = 0.71075 Å) was used. The structures were solved by direct methods with (SIR-97)[S4] and refined by full-matrix least-squares techniques against F^2(SHELXL-97).[S5] The intensities were corrected for Lorentz and polarization effects. Non-hydrogen atoms were refined anisotropically. Hydrogen atoms were placed using AFIX instructions. The data can be obtained free of charge from The Cambridge Crystallographic Data Centre via www.ccdc.cam.ac.uk/data_request/cif (CCDC 1045812).

Table S1: Crystallographic data and structure refinement details for compound ([$AuCl_3$(PyC)]·$PhNO_2$).

	[$AuCl_3$(PyC)]·$PhNO_2$
formula	$C_{72}H_{70}Au_2Cl_6N_4O_4$
fw	1661.95
T (K)	103(2)
λ (Å)	0.71075
Cryst syst	Monoclinic
space group	*C*2/*c*
a, (Å)	8.332(5)
b, (Å)	25.831(13)
c, (Å)	16.488(9)
α,□(deg □	90
β□(deg □	104.578(8)
γ,□(deg □	90
V, (Å^3)	3434(3)
Z	2
D_{calc}, (g / cm^3)	1.607
μ (mm^{-1})	4.551
F(000)	1644
cryst size (mm)	0.10 × 0.10 × 0.05
2θ range, (deg □	3.00–25.00
reflns collected	11449
indep reflns/R_{int}	3024/0.0567
params	312
GOF on F^2	1.043
R_1, wR_2 [I>2σ(I)]	0.0522, 0.1235
R_1, wR_2 (all data)	0.0676, 0.1320

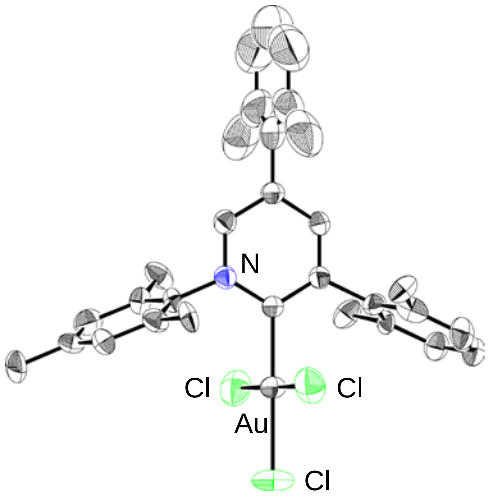

Figure S2: ORTEP drawing of $AuCl_3$(PyC) with 50% probability. All hydrogen atoms and nitrobenzene molecule are omitted for clarity.

Computational study

The Gaussian 09 program[S6] running on a SGI Altix4700 system was used for optimization (B3LYP/SDD[S7] for Au, 6-31G(d) for others). Structures were optimized without any symmetry assumptions. Zero-point energy, enthalpy, and Gibbs free energy at 298.15 K and 1 atm were estimated from the gas-phase studies. Harmonic vibration frequency calculation at the same level was performed to verify all stationary points as local minima (with no imaginary frequency). Visualization of the results was performed by use of GaussView 5.0.9 software.

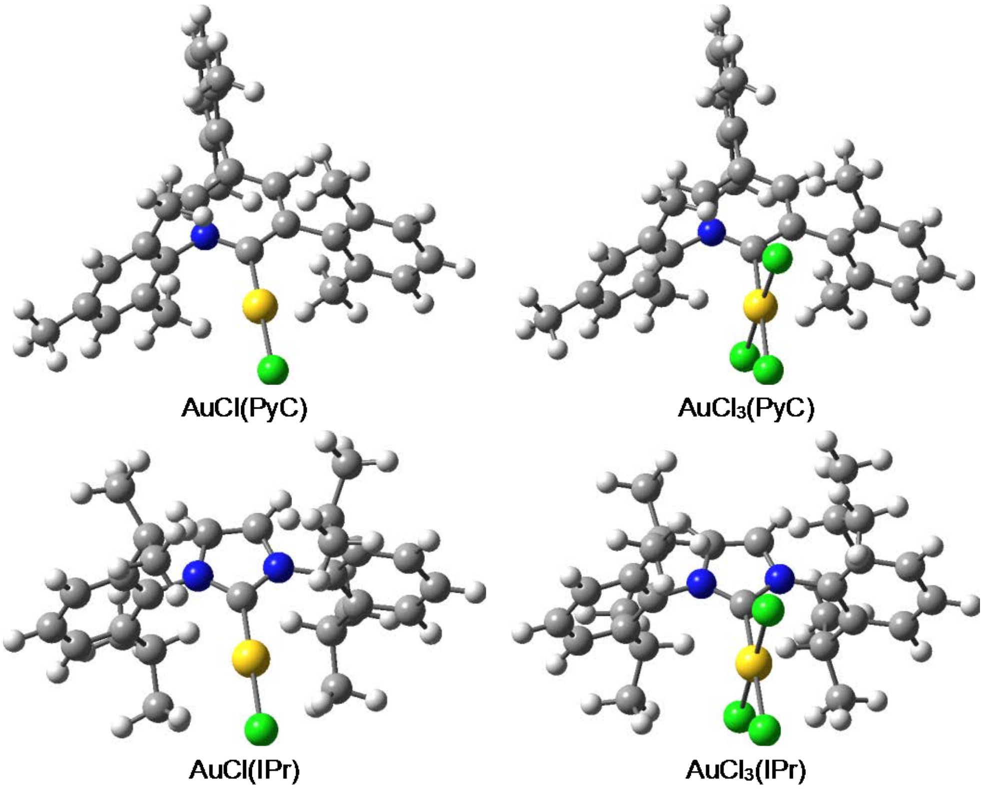

Figure S3: Optimized structures of gold complexes. C: gray, H: white, N: blue, Cl: green, Au: yellow.

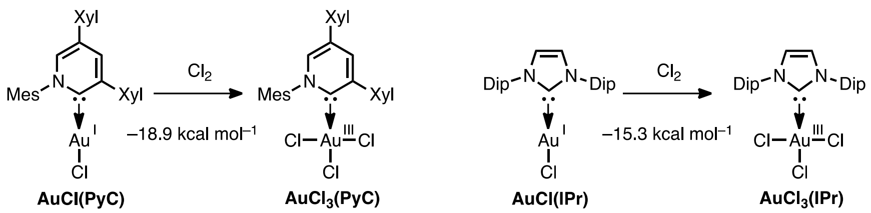

Scheme S1: Hypothetical reactions for estimation of the stability of gold(III) complexes.

Table S2: Uncorrected and thermal-corrected (298 K) energies of stationary points (Hartree).[a]

compound	*E*	*E* + *ZPE*	*H*	*G*
AuCl(PyC)	-1812.67004697	-1812.142233	-1812.106688	-1812.213402
$AuCl_3$(PyC)	-2733.05219302	-2732.520790	-2732.482012	-2732.594673
AuCl(IPr)	-1756.11982458	-1755.544370	-1755.509197	-1755.614045
$AuCl_3$(PyC)	-2676.49630661	-2675.917036	-2675.878847	-2675.988002
Cl_2	-920.349884457	-920.348706	-920.345190	-920.370557

a) *E*: electronic energy; *ZPE*: zero-point energy; *H* (= $E + ZPE + E_{vib} + E_{rot} + E_{trans} + RT$): sum of

electronic and thermal enthalpies; *G* (= *H* – *TS*): sum of electronic and thermal free energies.

Table S3: Cartesian coordinates for the gold complexes and chlorine.

AuCl(PyC)

C	-0.686338	-1.334196	0.000074	H	1.223266	-6.365996	0.000263	H	-1.171639	-2.353408	2.657842
C	-2.063553	-1.144797	-0.000006	C	-4.120086	0.360864	0.000049	H	0.084655	-3.365845	3.384284
C	-2.638692	0.143335	0.000055	C	-4.811639	0.454251	1.226947	C	-0.134329	-2.693286	-2.549550
C	-1.760980	1.205133	0.000144	C	-4.811722	0.453554	-1.226855	H	-1.172417	-2.354162	-2.657585
H	-2.719914	-2.011902	-0.000100	C	-6.198247	0.645302	1.205664	H	0.507452	-1.810153	-2.656077
H	-2.089884	2.237721	0.000232	C	-6.198330	0.644602	-1.205588	H	0.084088	-3.366350	-3.383990
C	0.409028	2.230102	0.000026	C	-6.889334	0.740583	0.000034	C	-4.086105	0.353314	-2.550789
C	0.766753	2.800192	1.230823	H	-6.737181	0.717838	2.146908	H	-3.366363	1.170655	-2.684973
C	0.766391	2.800180	-1.230884	H	-6.737329	0.716591	-2.146838	H	-3.520644	-0.582125	-2.638817
C	1.518795	3.978262	1.200578	H	-7.965994	0.887549	0.000028	H	-4.792731	0.395463	-3.384743
C	1.518447	3.978246	-1.200873	N	-0.407771	1.016310	0.000114	C	-4.085965	0.354556	2.550894
C	1.911466	4.577842	-0.000209	C	0.389926	2.161686	-2.546114	H	-3.521128	-0.581212	2.639525
H	1.810675	4.433367	2.144205	H	0.893399	1.194853	-2.667121	H	-3.365665	1.171498	2.684440
H	1.810074	4.433329	-2.144589	H	-0.688816	1.980539	-2.627991	H	-4.792494	0.397757	3.384875
C	-0.137436	-2.730375	0.000118	H	0.685856	2.801438	-3.382015	C	2.761353	5.826604	-0.000327
C	0.105071	-3.384958	-1.226089	C	0.390723	2.161702	2.546177	H	3.828730	5.570546	-0.000019
C	0.105420	-3.384732	1.226380	H	-0.687881	1.979793	2.628101	H	2.573297	6.440327	-0.887504
C	0.594564	-4.695556	-1.204923	H	0.894884	1.195244	2.667343	H	2.572900	6.440763	0.886467
C	0.594924	-4.695324	1.205313	H	0.686215	2.801813	3.381960	C	0.200868	-0.217526	0.000078
C	0.840058	-5.349138	0.000220	C	-0.133652	-2.692825	2.549779	Au	2.211308	-0.383949	-0.000056
H	0.788817	-5.202537	-2.146734	H	0.508379	-1.809854	2.656137	Cl	4.532043	-0.589953	-0.000229
H	0.789469	-5.202121	2.147162								

$AuCl_3$(PyC)

C	-0.852015	-1.320809	0.075220	H	1.067140	-6.348485	0.230580	H	0.046875	-3.236463	3.520291
C	-2.238233	-1.150297	0.076545	C	-4.326226	0.303783	-0.003606	C	-0.782144	-2.952272	-2.380877
C	-2.841206	0.115626	0.007800	C	-5.028008	0.369038	1.219276	H	-1.876528	-2.850769	-2.364795
C	-1.985339	1.196166	-0.048046	C	-5.005699	0.400445	-1.236872	H	-0.356182	-1.976350	-2.625778
H	-2.871164	-2.032480	0.126879	C	-6.417094	0.537803	1.186756	H	-0.531979	-3.637401	-3.196176
H	-2.336620	2.219471	-0.098495	C	-6.395496	0.567326	-1.224948	C	-4.267130	0.326080	-2.555492
C	0.136077	2.301160	-0.060553	C	-7.098251	0.636636	-0.024329	H	-3.578727	1.170340	-2.688493
C	0.344343	2.971083	1.156642	H	-6.965650	0.590734	2.123590	H	-3.666113	-0.587136	-2.639377
C	0.524491	2.837636	-1.299853	H	-6.927453	0.641339	-2.169898	H	-4.969379	0.340863	-3.393852
C	1.087975	4.154182	1.114619	H	-8.177052	0.766030	-0.032440	C	-4.312004	0.262330	2.547952
C	1.261904	4.026001	-1.275357	N	-0.629499	1.045245	-0.038343	H	-3.792060	-0.697377	2.657842
C	1.581455	4.681292	-0.082386	C	0.123979	2.220088	-2.617541	H	-3.554720	1.046365	2.670265
H	1.281820	4.676784	2.048038	H	0.590440	1.242719	-2.771255	H	-5.019123	0.351807	3.377476
H	1.586915	4.451480	-2.221603	H	-0.963683	2.084743	-2.683420	C	2.432057	5.928644	-0.087696
C	-0.315614	-2.723528	0.126273	H	0.425893	2.869408	-3.443810	H	3.496452	5.670134	-0.017015
C	-0.259960	-3.483640	-1.064585	C	-0.261883	2.504697	2.459230	H	2.296604	6.503721	-1.009648
C	0.029981	-3.302000	1.368541	H	-1.336455	2.734279	2.495389	H	2.196780	6.579322	0.760879
C	0.250784	-4.784941	-1.004780	H	-0.132433	1.432325	2.616436	C	-0.015040	-0.177904	0.013574
C	0.532901	-4.608560	1.380718	H	0.209743	3.018151	3.301168	Au	2.061010	-0.308670	-0.024243
C	0.664760	-5.339447	0.203440	C	-0.182246	-2.576768	2.678069	Cl	2.014002	-0.782012	-2.337745
H	0.317519	-5.366349	-1.920528	H	0.454052	-1.691382	2.768319	Cl	2.201595	0.132851	2.290896
H	0.814870	-5.053809	2.331181	H	-1.224321	-2.248832	2.790435	Cl	4.417258	-0.475197	-0.085212

AuCl(IPr)

C	0.678108	0.000225	-2.656979	H	1.370313	2.383433	-1.187702	H	-1.370154	-2.383222	-1.187814
C	-0.678047	0.000267	-2.656990	C	2.411053	-2.578079	-0.911683	C	-3.044255	3.389541	-2.059311
H	1.392082	0.000311	-3.465262	H	1.369950	-2.383252	-1.187141	H	-3.024100	2.833497	-3.003849
H	-1.392006	0.000392	-3.465286	C	3.044760	3.389526	-2.059394	H	-4.089111	3.641219	-1.843818
C	2.465223	0.000054	-0.907902	H	4.089545	3.641236	-1.843596	H	-2.499657	4.329510	-2.206439

C	3.113445	-1.237086	-0.723446	H	3.024895	2.833521	-3.003962	C	-2.387480	3.389925	0.398843
C	3.113579	1.237126	-0.723516	H	2.500179	4.329485	-2.206642	H	-3.399796	3.652265	0.727671
C	4.461085	-1.206400	-0.344884	C	2.387319	3.389782	0.398585	H	-1.905887	2.825702	1.204116
C	4.461215	1.206314	-0.344948	H	1.905469	2.825532	1.203685	H	-1.832556	4.324434	0.253712
C	5.129930	-0.000073	-0.157312	H	3.399558	3.652040	0.727717	C	-3.044511	-3.389128	-2.059926
H	4.990671	-2.141817	-0.189795	H	1.832483	4.324330	0.253352	H	-4.089336	-3.640827	-1.844305
H	4.990901	2.141681	-0.189895	C	3.044055	-3.389102	-2.059824	H	-3.024510	-2.832951	-3.004391
H	6.175154	-0.000119	0.139814	H	3.023758	-2.832852	-3.004240	H	-2.499950	-4.329082	-2.207282
C	-2.465192	0.000127	-0.907944	H	4.088952	-3.640781	-1.844524	C	-2.387366	-3.389878	0.398126
C	-3.113471	1.237216	-0.723382	H	2.499471	-4.329056	-2.207089	H	-1.832502	-4.324389	0.252759
C	-3.113498	-1.236998	-0.723678	C	2.387587	-3.390065	0.398406	H	-1.905610	-2.825791	1.203398
C	-4.461114	1.206435	-0.344839	H	3.399933	-3.652521	0.727045	H	-3.399635	-3.652223	0.727087
C	-4.461141	-1.206278	-0.345131	H	1.906101	-2.826031	1.203876	Au	-0.000019	-0.000133	1.517572
C	-5.129911	0.000064	-0.157393	H	1.832621	-4.324525	0.253111	Cl	-0.000099	-0.000367	3.842650
H	-4.990743	2.141813	-0.189656	C	-2.411112	2.578253	-0.911437	N	1.079889	0.000124	-1.326043
H	-4.990790	-2.141684	-0.190181	H	-1.370049	2.383480	-1.187092	N	-1.079850	0.000166	-1.326062
H	-6.175140	0.000042	0.139716	C	-2.411180	-2.578013	-0.912037	C	0.000013	0.000052	-0.491192
C	2.411300	2.578181	-0.911739								

$AuCl_3(IPr)$

C	0.676288	-0.032413	-2.676852	H	1.546429	2.390487	-1.189370	C	-2.564123	3.236548	-2.353896
C	-0.676580	0.034421	-2.676754	C	2.313678	-2.686690	-0.934076	H	-2.257962	2.524276	-3.128668
H	1.388988	-0.069739	-3.484013	H	1.243889	-2.486153	-0.833621	H	-3.627297	3.457557	-2.506309
H	-1.389357	0.072437	-3.483817	C	2.963464	2.792192	-2.759804	H	-2.000678	4.164136	-2.508754
C	2.489332	-0.109616	-0.961815	H	4.047090	2.868167	-2.908741	C	-2.656353	3.754264	0.122723
C	3.070649	-1.375886	-0.735608	H	2.585945	2.034733	-3.455622	H	-3.684329	4.121087	0.019778
C	3.224490	1.094897	-0.912507	H	2.516747	3.754357	-3.036392	H	-2.520785	3.369791	1.137155
C	4.428314	-1.400993	-0.392531	C	3.101807	3.592973	-0.368029	H	-1.990499	4.615385	0.000318
C	4.578159	0.999849	-0.566920	H	2.928280	3.351574	0.683453	C	-2.964528	-2.789623	-2.761675
C	5.172085	-0.229204	-0.298546	H	4.165234	3.821037	-0.505474	H	-4.048189	-2.865154	-2.910608
H	4.908976	-2.354358	-0.201374	H	2.542705	4.505630	-0.602836	H	-2.586813	-2.031774	-3.456964
H	5.174591	1.903993	-0.508154	C	2.564797	-3.234503	-2.356404	H	-2.518126	-3.751726	-3.038983
H	6.222924	-0.274866	-0.025741	H	2.258373	-2.521784	-3.130662	C	-3.102999	-3.592135	-0.370512
C	-2.489443	0.110696	-0.961518	H	3.628020	-3.455102	-2.509067	H	-2.544163	-4.504775	-0.606015
C	-3.070390	1.376965	-0.734334	H	2.001606	-4.162146	-2.511851	H	-2.929387	-3.351561	0.681146
C	-3.224945	-1.093638	-0.913109	C	2.657435	-3.753881	0.119850	H	-4.166496	-3.819788	-0.508099
C	-4.428028	1.402202	-0.391171	H	3.685542	-4.120242	0.016580	Au	0.000085	-0.000912	1.520837
C	-4.578577	-0.998461	-0.567407	H	2.521797	-3.370164	1.134558	Cl	0.348714	2.328705	1.525695
C	-5.172128	0.230555	-0.298040	H	1.991895	-4.615167	-0.003109	Cl	0.000195	-0.002157	3.871312
H	-4.908413	2.355558	-0.199278	C	-2.313048	2.687685	-0.931963	Cl	-0.348559	-2.330541	1.523151
H	-5.175270	-1.902478	-0.509320	H	-1.243312	2.486756	-0.831741	N	1.085765	-0.049276	-1.348370
H	-6.222943	0.276315	-0.025156	C	-2.634644	-2.450538	-1.291019	N	-1.085925	0.050288	-1.348217
C	2.633771	2.451915	-1.289376	H	-1.547279	-2.389487	-1.191023	C	-0.000036	0.000100	-0.534522

Cl_2

Cl	0.0	0.0	1.021364	Cl	0.0	0.0	-1.021364

References

S1) K. Hata, Y. Segawa, K. Itami, *Chem. Commun.* **2012**, *48*, 6642.

S2) S. Gaillard, A. M. Z. Slawin, A. T. Bonura, E. D. Stevens, S. T. Nolan, *Organometallics* **2010**, *29*, 394.

S3) K. Funaki, T. Sato, S. Oi, *Org. Lett.* **2012**, *14*, 6186.

S4) A. Altomare, M. C. Burla, M. Camalli, G. L. Cascarano, C. Giacovazza, A. Guagliardi, A. G. G. Moliterni, G. Polidori, R. Spagna, *J. Appl. Crystallogr*. **1999**, *32*, 115.

S5) G. M. Sheldrick, University of Göttingen: Göttingen, Germany **1997**.

S6) M. J. Frisch, G. W. Trucks, H. B. Schlegel, G. E. Scuseria, M. A. Robb, J. R. Cheeseman, G. Scalmani, V. Barone, B. Mennucci, G. A. Petersson, H. Nakatsuji, M. Caricato, X. Li, H. P. Hratchian, A. F. Izmaylov, J. Bloino, G. Zheng, J. L. Sonnenberg, M. Hada, M. Ehara, K. Toyota, R. Fukuda, J. Hasegawa, M. Ishida, T. Nakajima, Y. Honda, O. Kitao, H. Nakai, T. Vreven, J. A. Montgomery, Jr., J. E. Peralta, F. Ogliaro, M. Bearpark, J. J. Heyd, E. Brothers, K. N. Kudin, V. N. Staroverov, R. Kobayashi, J. Normand, K. Raghavachari, A. Rendell, J. C. Burant, S. S. Iyengar, J. Tomasi, M. Cossi, N. Rega, J. M. Millam, M. Klene, J. E. Knox, J. B. Cross, V. Bakken, C. Adamo, J. Jaramillo, R. Gomperts, R. E. Stratmann, O. Yazyev, A. J. Austin, R. Cammi, C. Pomelli, J. W. Ochterski, R. L. Martin, K. Morokuma, V. G. Zakrzewski, G. A. Voth, P. Salvador, J. J. Dannenberg, S. Dapprich, A. D. Daniels, O. Farkas, J. B. Foresman, J. V. Ortiz, J. Cioslowski, D. J. Fox, Gaussian 09, Revision D.01, Gaussian, Inc., Wallingford CT, **2013**.

S7) P. Fuentealba, H. Preuss, H. Stoll, L. Von Szentpály, *Chem. Phys. Lett.* **1982**, *89*, 418.

^{1}H and ^{13}C NMR spectra

^{1}H NMR spectrum of $AuCl_3(PyC)$ (600 MHz, $CDCl_3$)

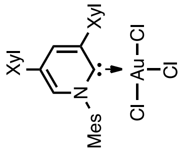

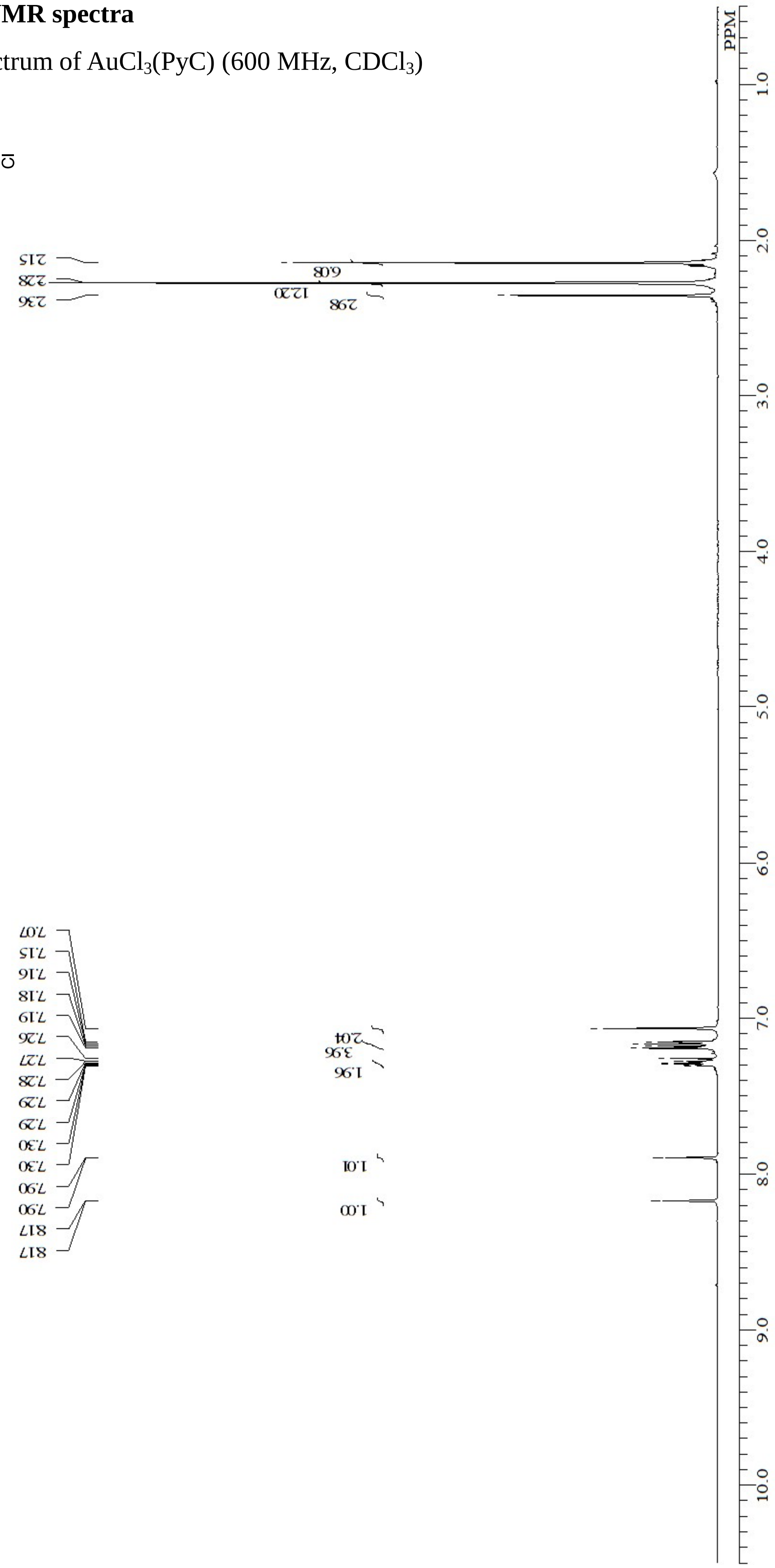

^{13}C NMR spectrum of $AuCl_3$(PyC) (150 MHz, $CDCl_3$)

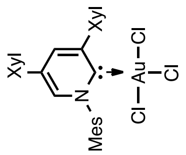

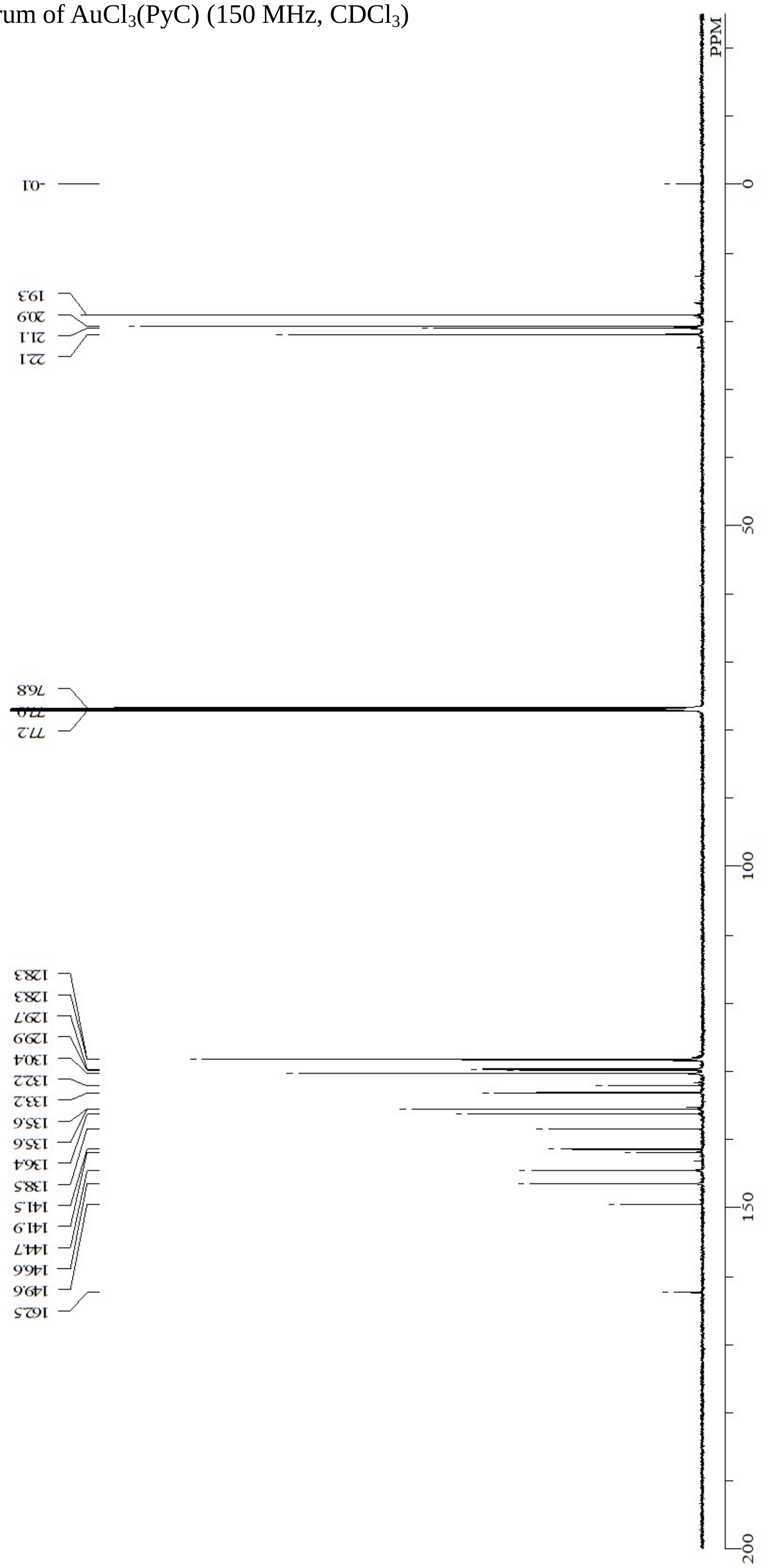

^{1}H NMR spectrum of **3aa** (600 MHz, $CDCl_3$)

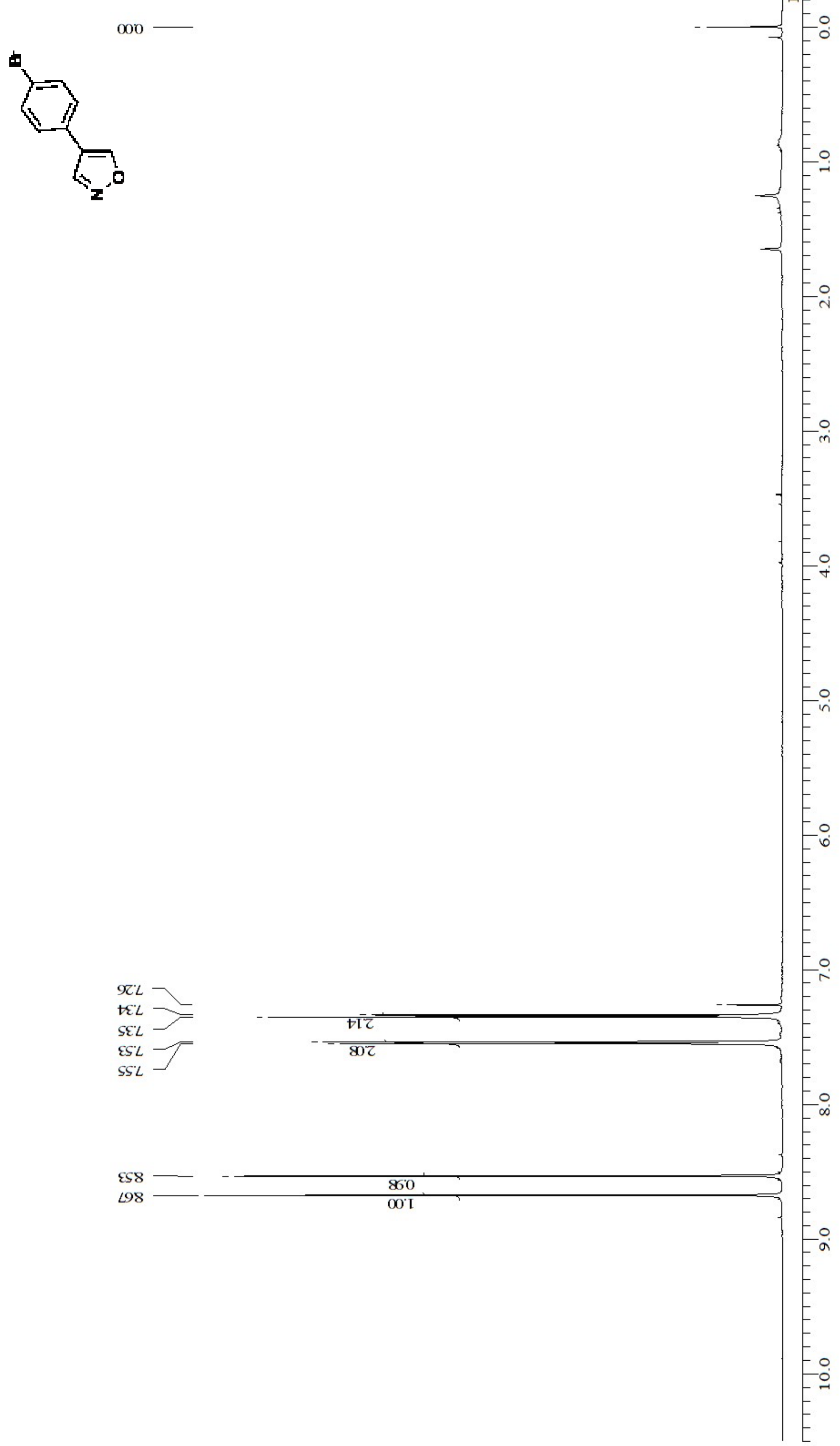

^{13}C NMR spectrum of **3aa** (150 MHz, $CDCl_3$)

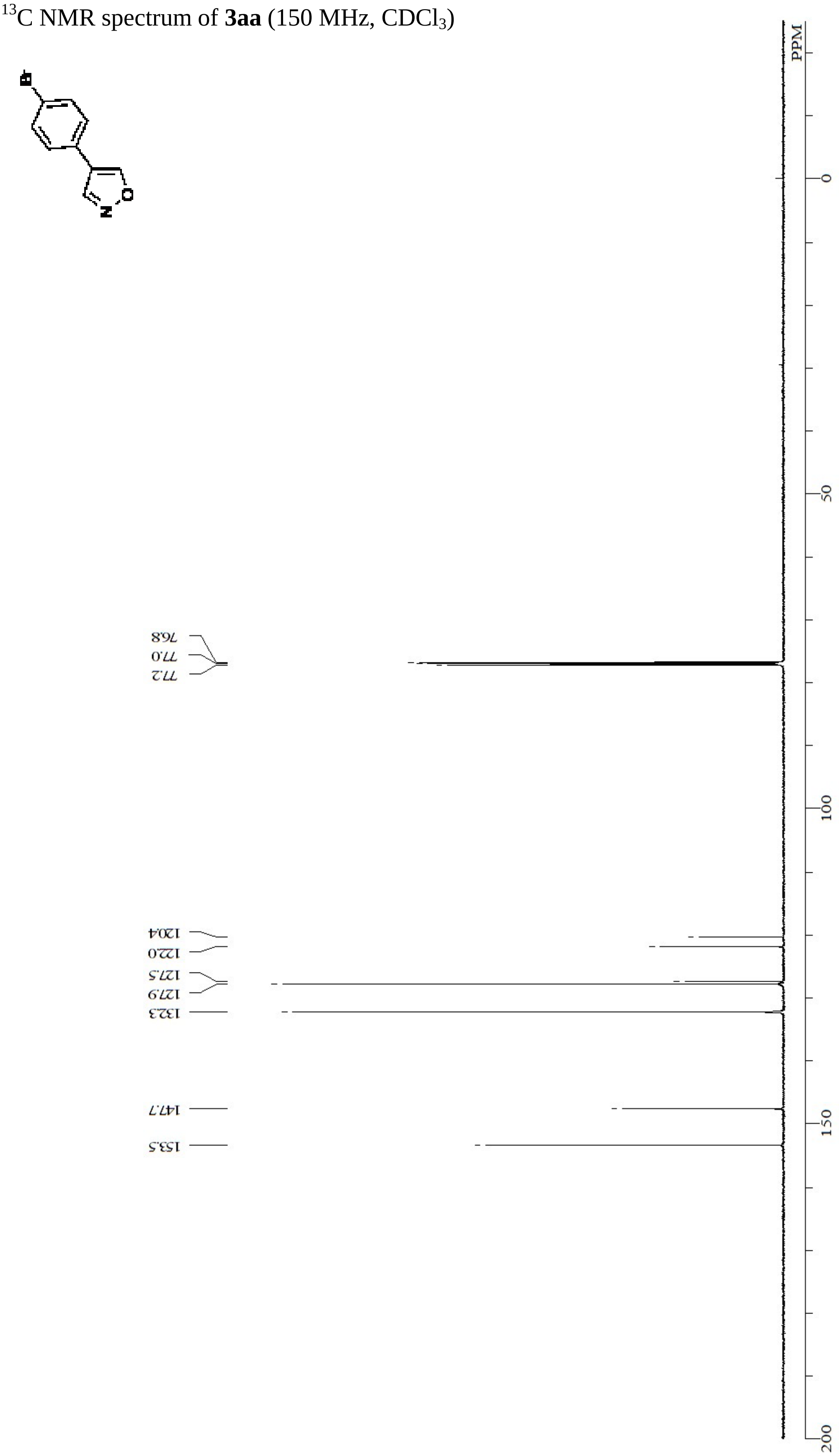

^{1}H NMR spectrum of **3ab** (600 MHz, $CDCl_3$)

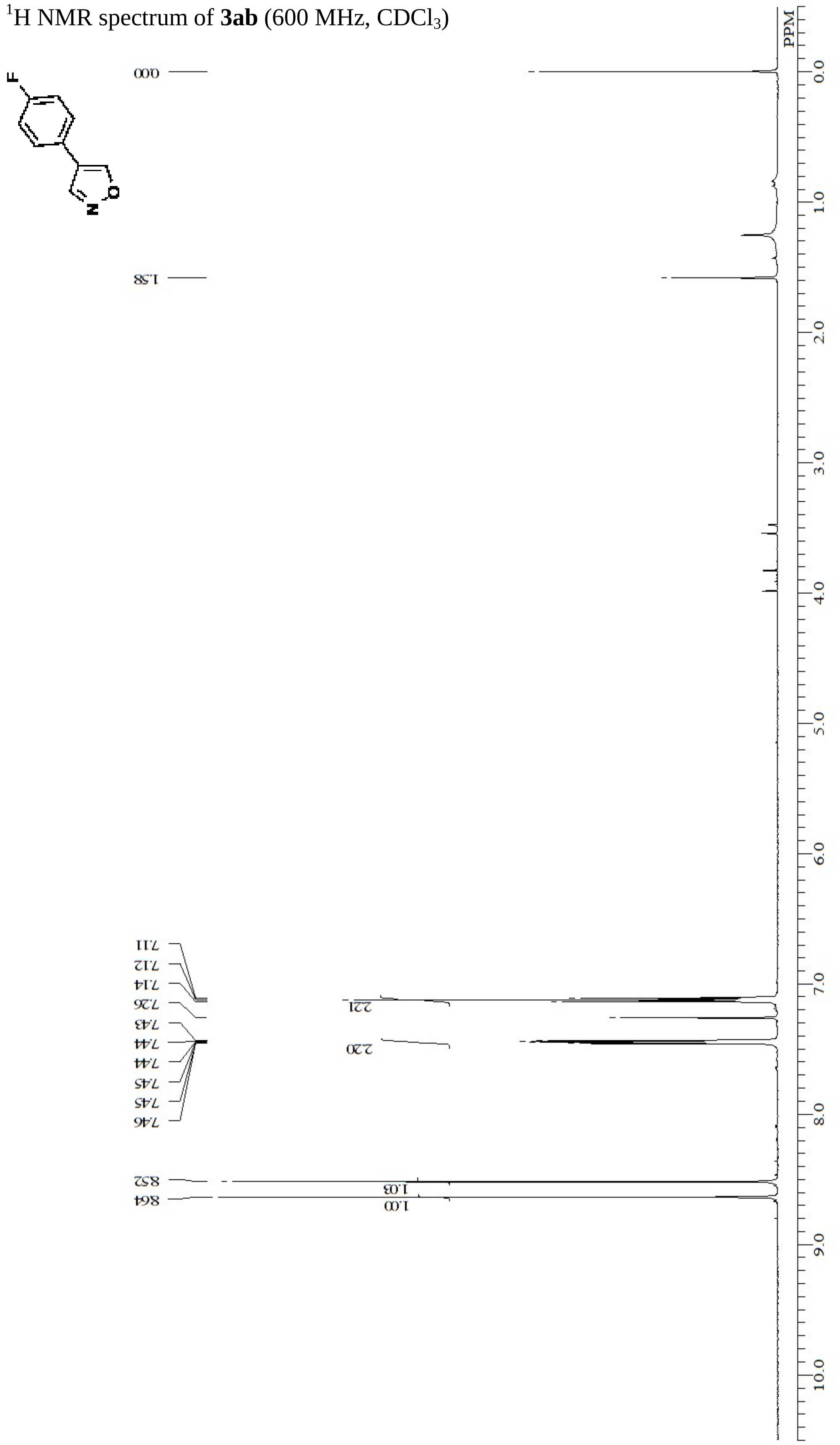

^{13}C NMR spectrum of **3ab** (150 MHz, $CDCl_3$)

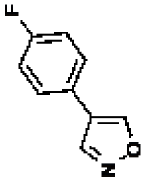

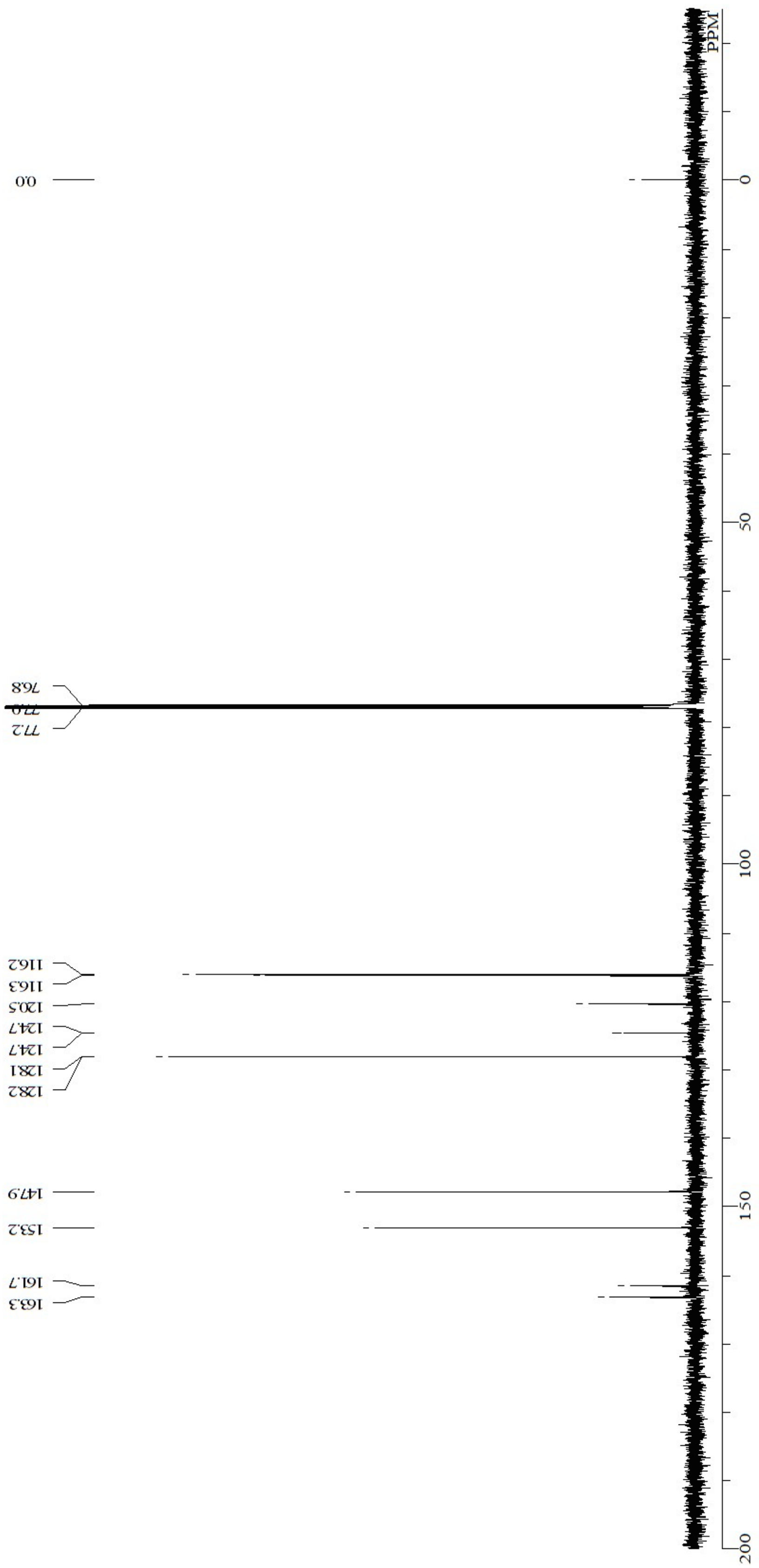

^{1}H NMR spectrum of **3ba** (600 MHz, $CDCl_3$)

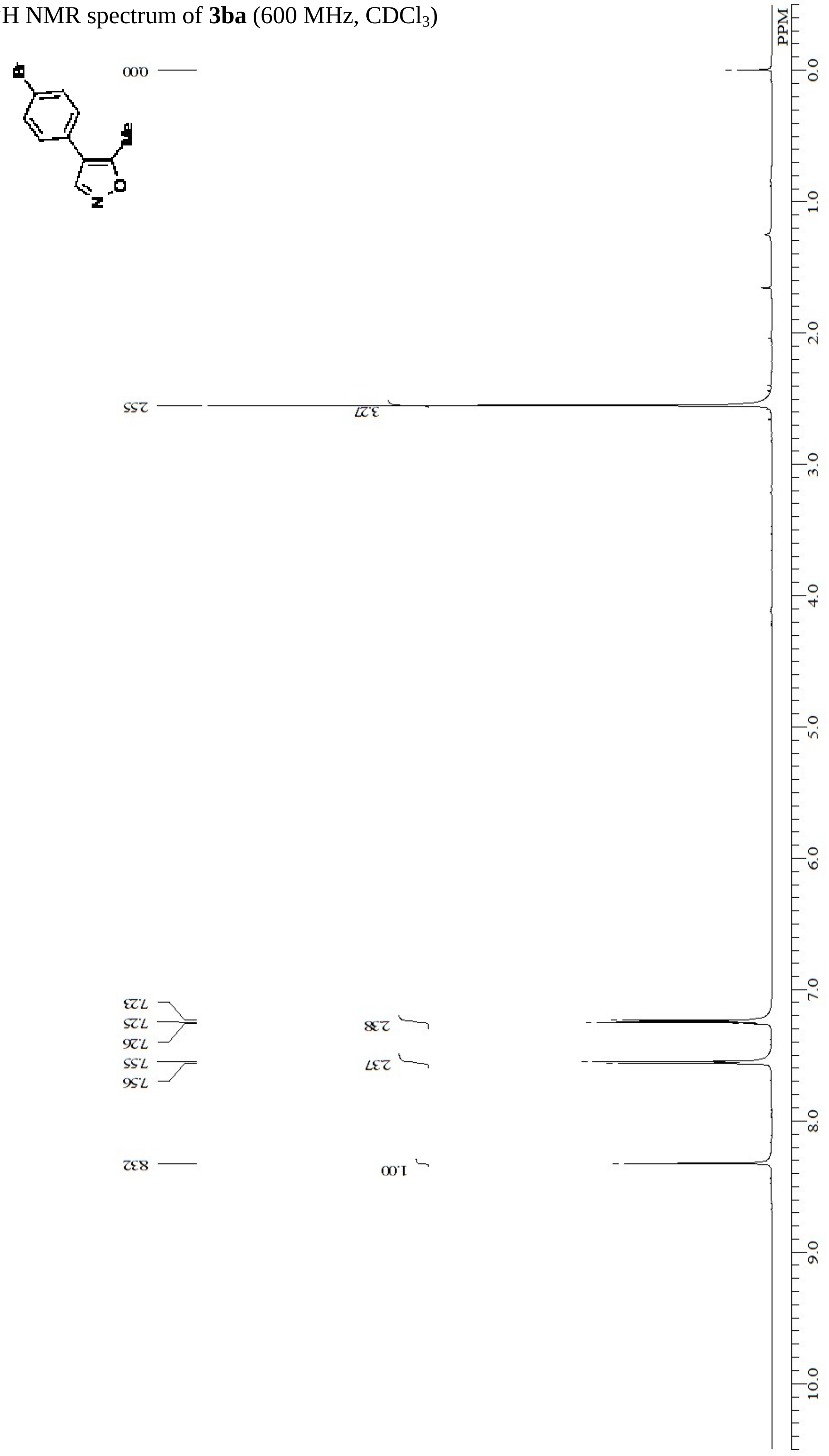

^{13}C NMR spectrum of **3ba** (150 MHz, $CDCl_3$)

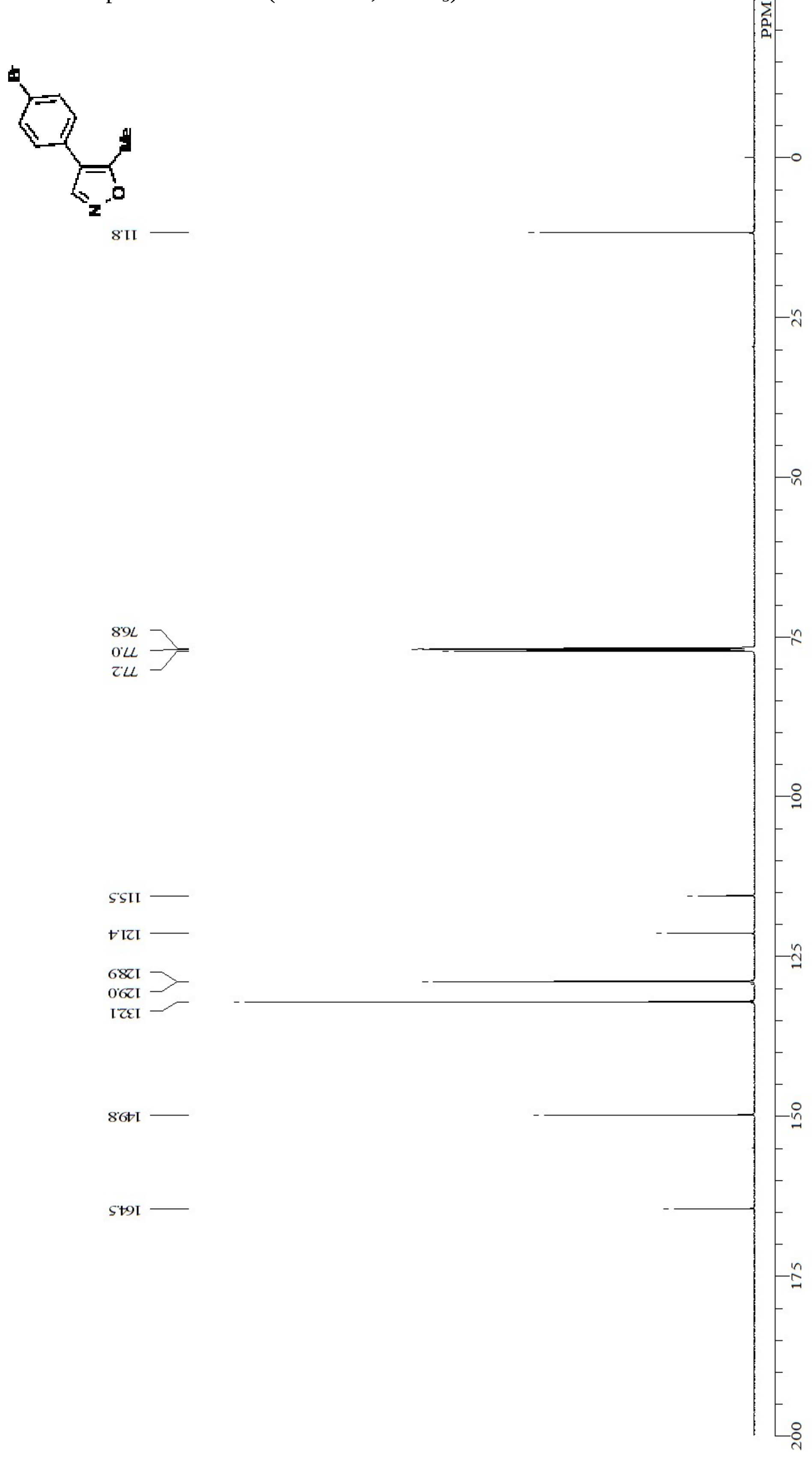

^{1}H NMR spectrum of **3bb** (600 MHz, $CDCl_3$)

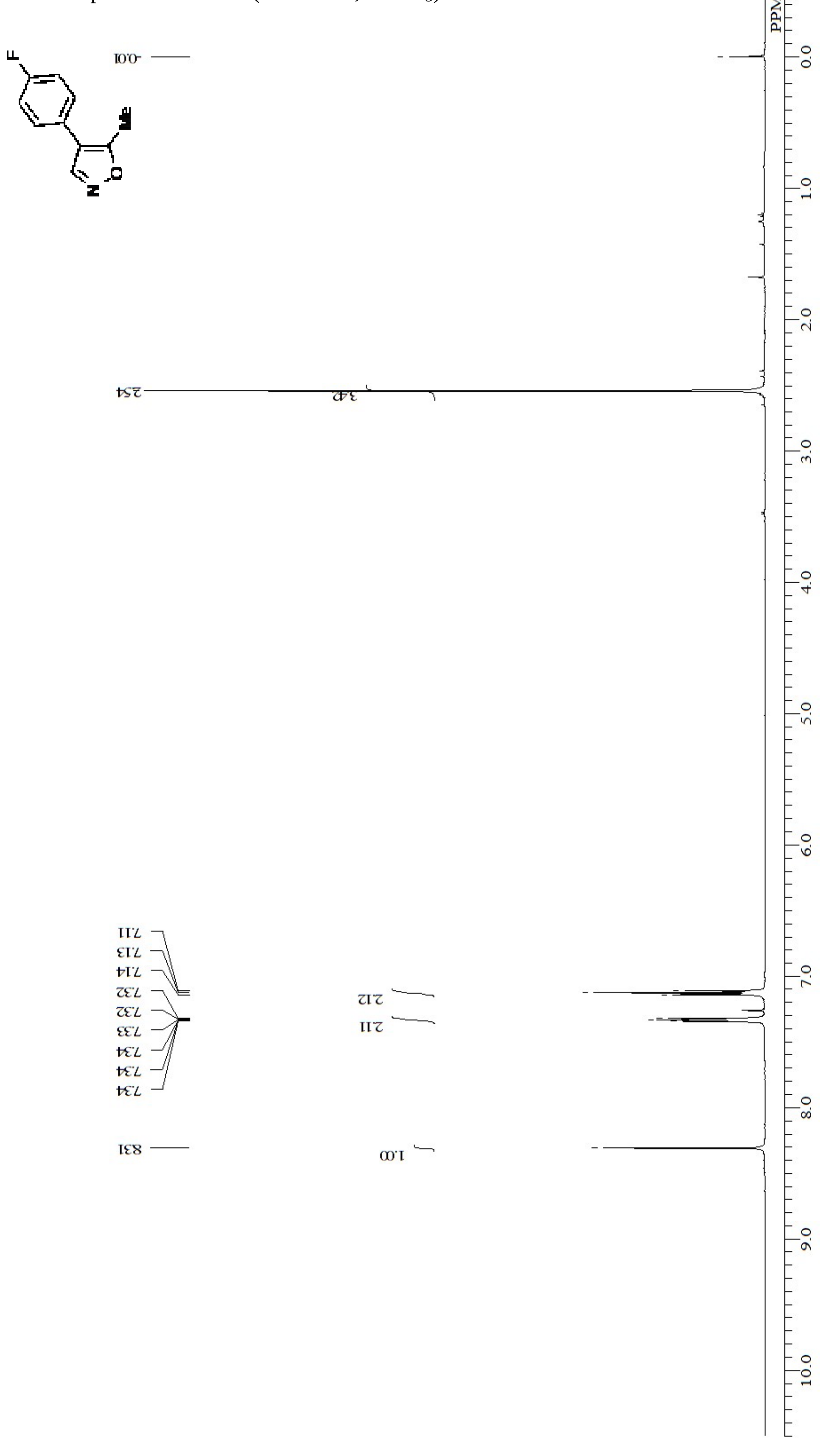

^{13}C NMR spectrum of **3bb** (150 MHz, $CDCl_3$)

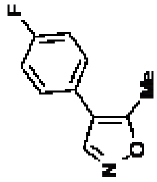

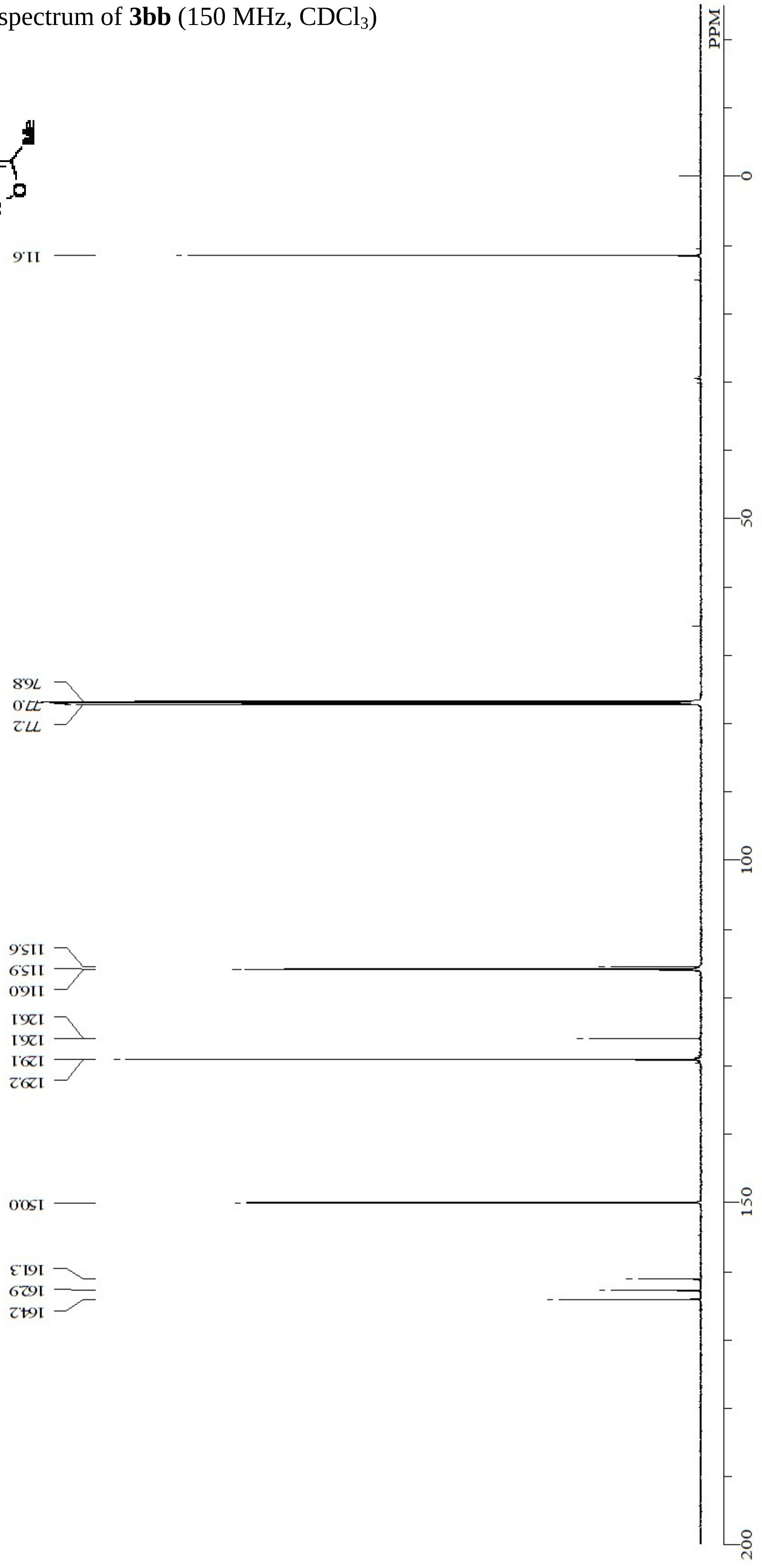

^{1}H NMR spectrum of **3bc** (600 MHz, $CDCl_3$)

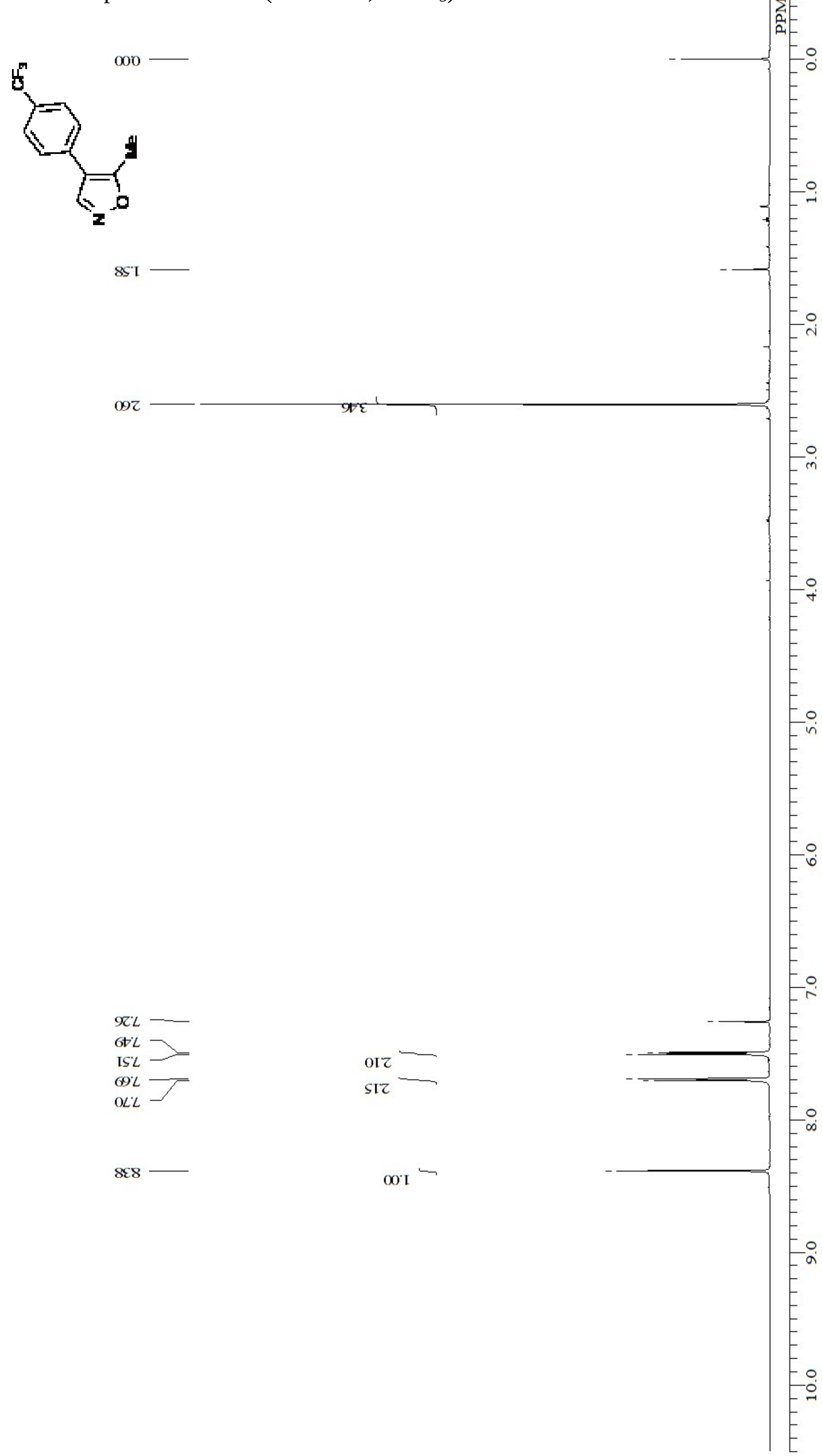

^{13}C NMR spectrum of **3bc** (150 MHz, $CDCl_3$)

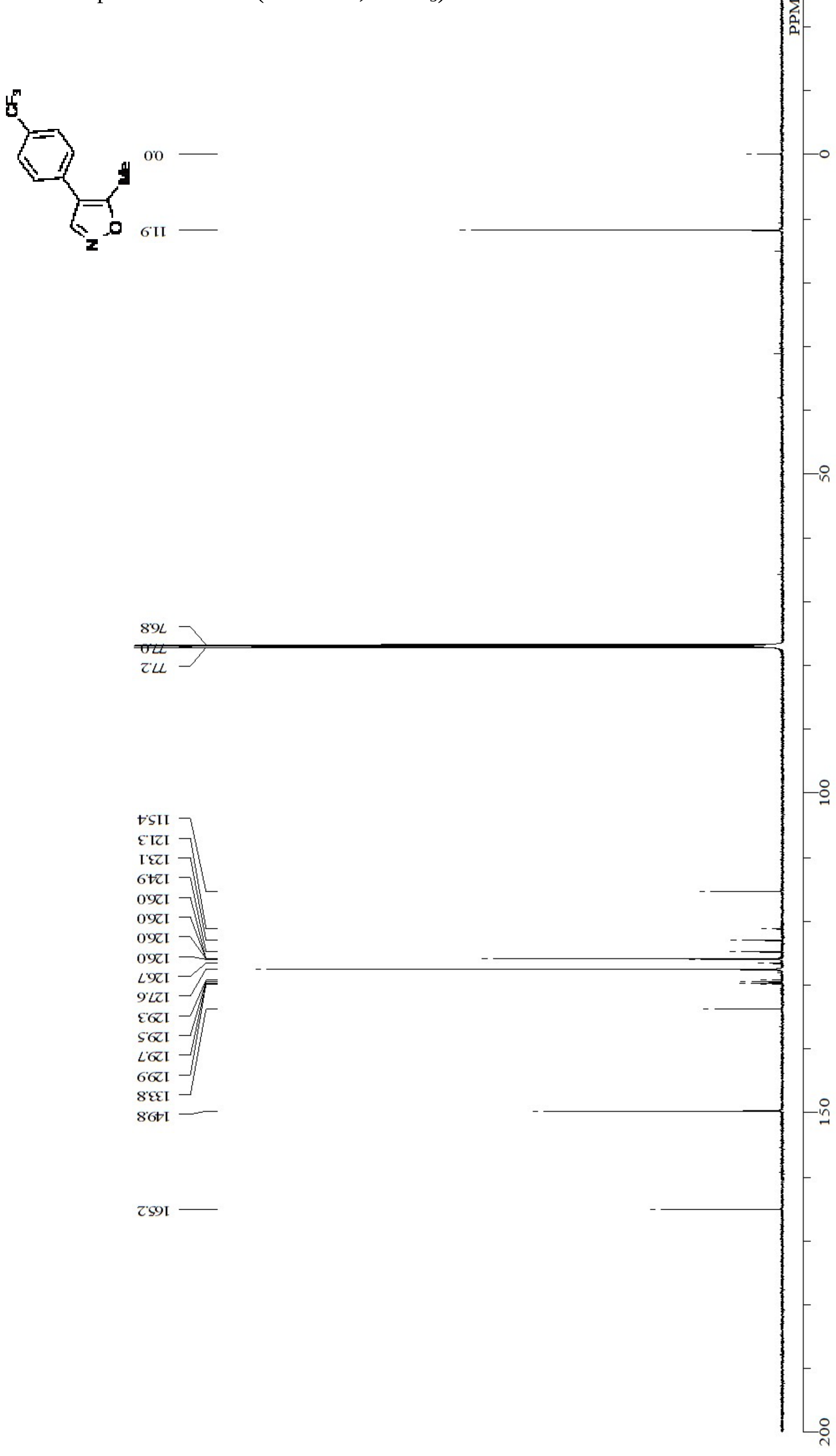

^{1}H NMR spectrum of **3bd** (600 MHz, $CDCl_3$)

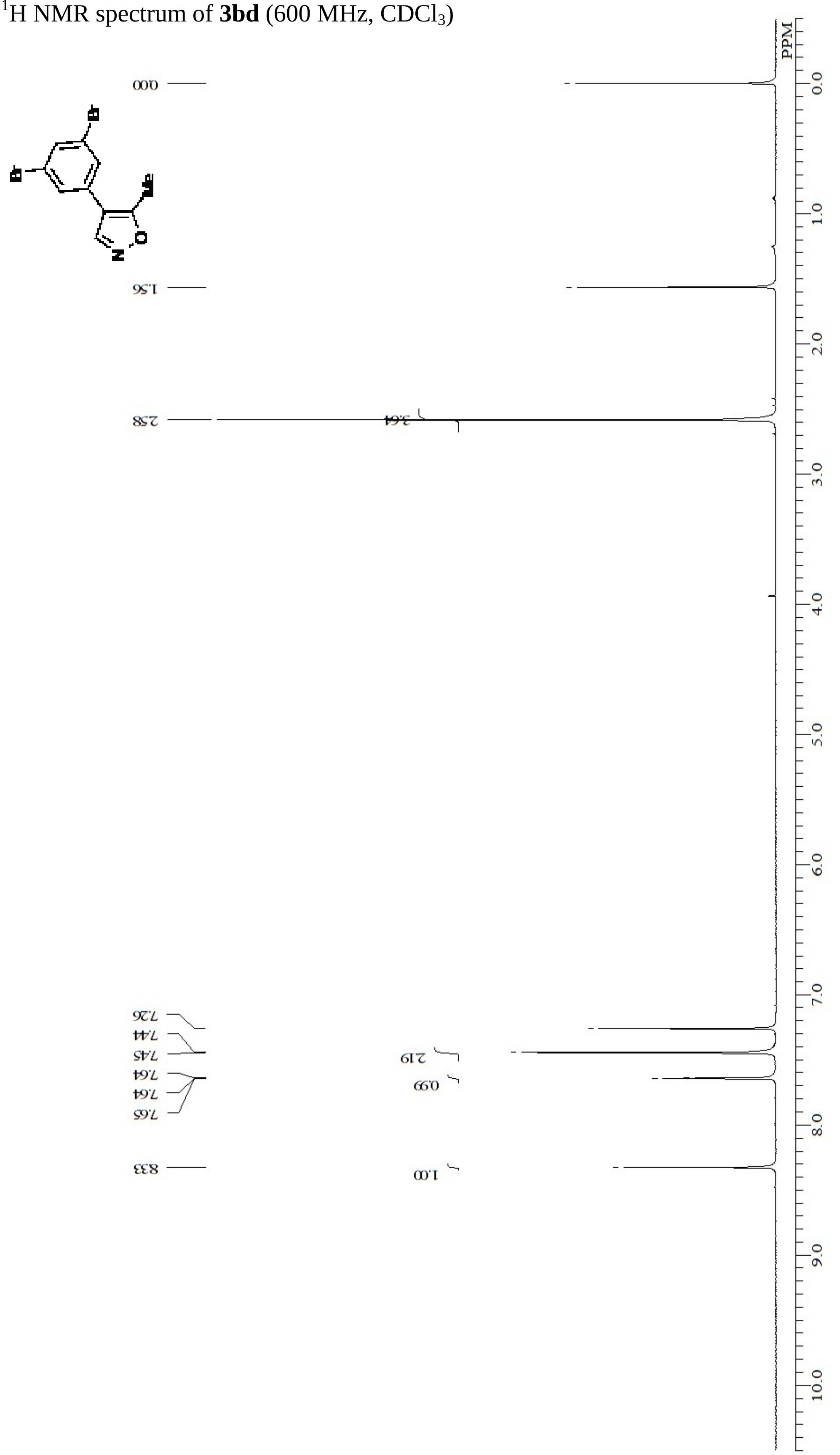

^{13}C NMR spectrum of **3bd** (150 MHz, $CDCl_3$)

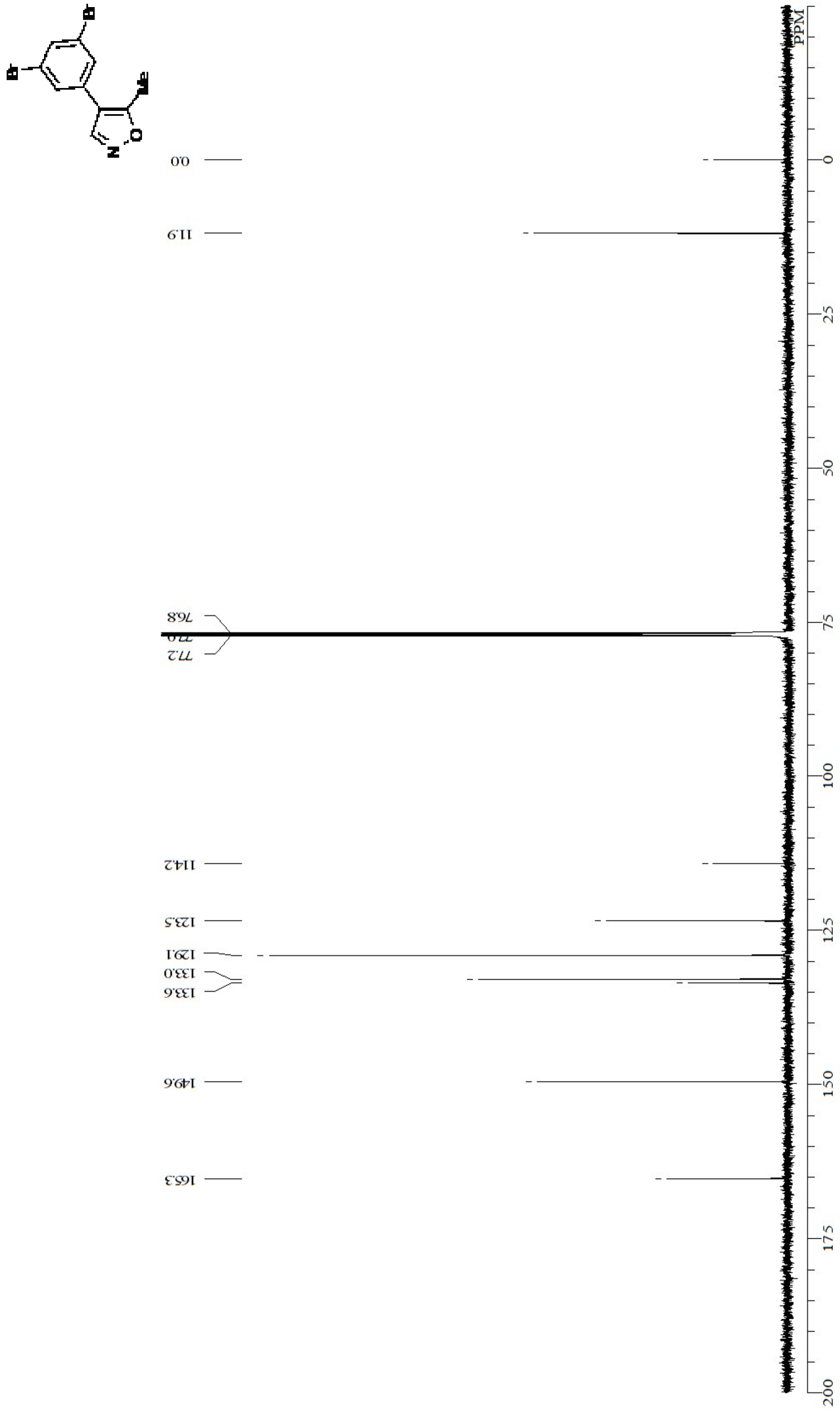

^{1}H NMR spectrum of **3ca** (600 MHz, $CDCl_3$)

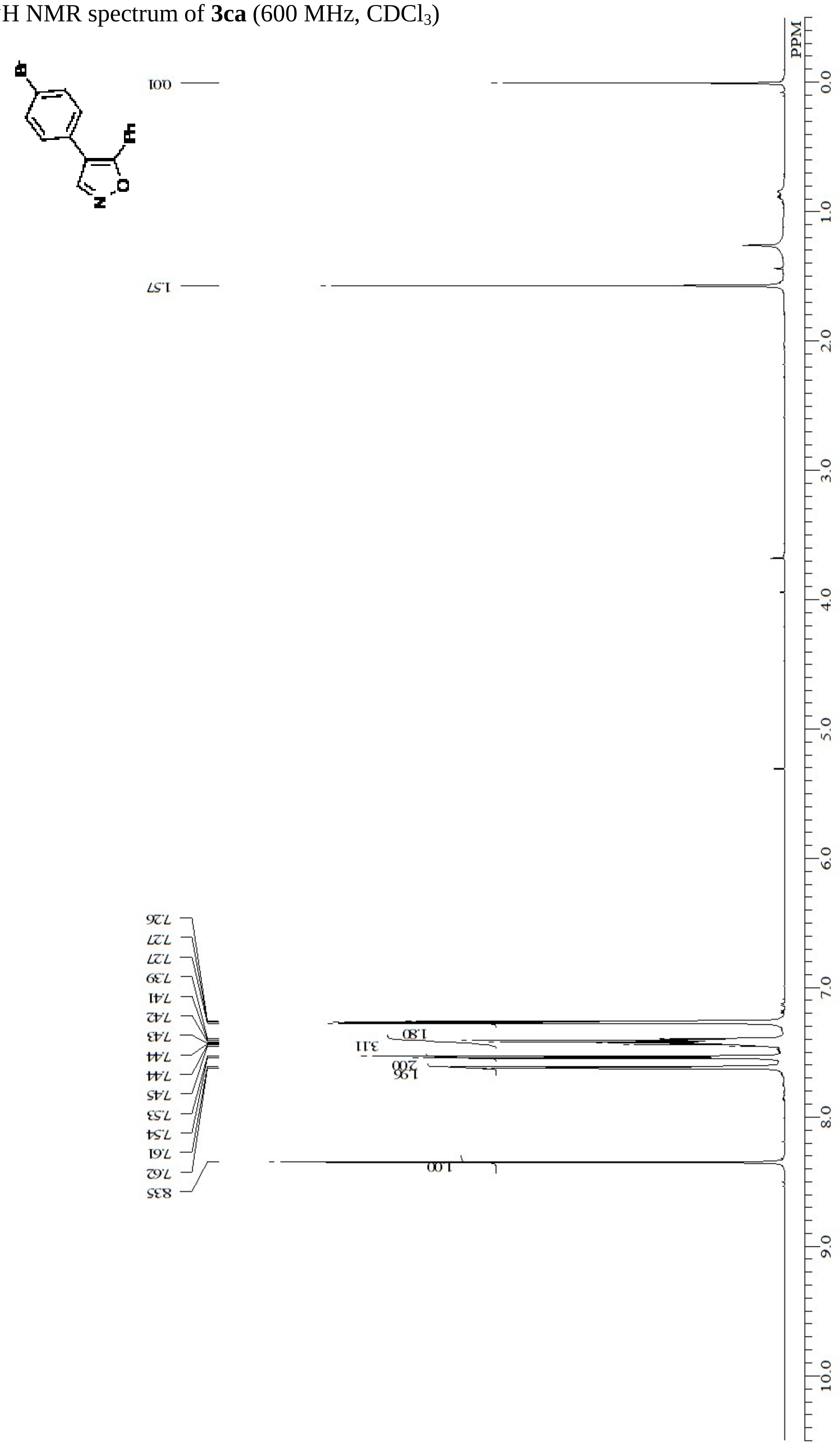

^{13}C NMR spectrum of **3ca** (150 MHz, $CDCl_3$)

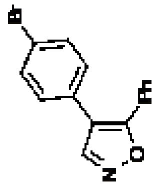

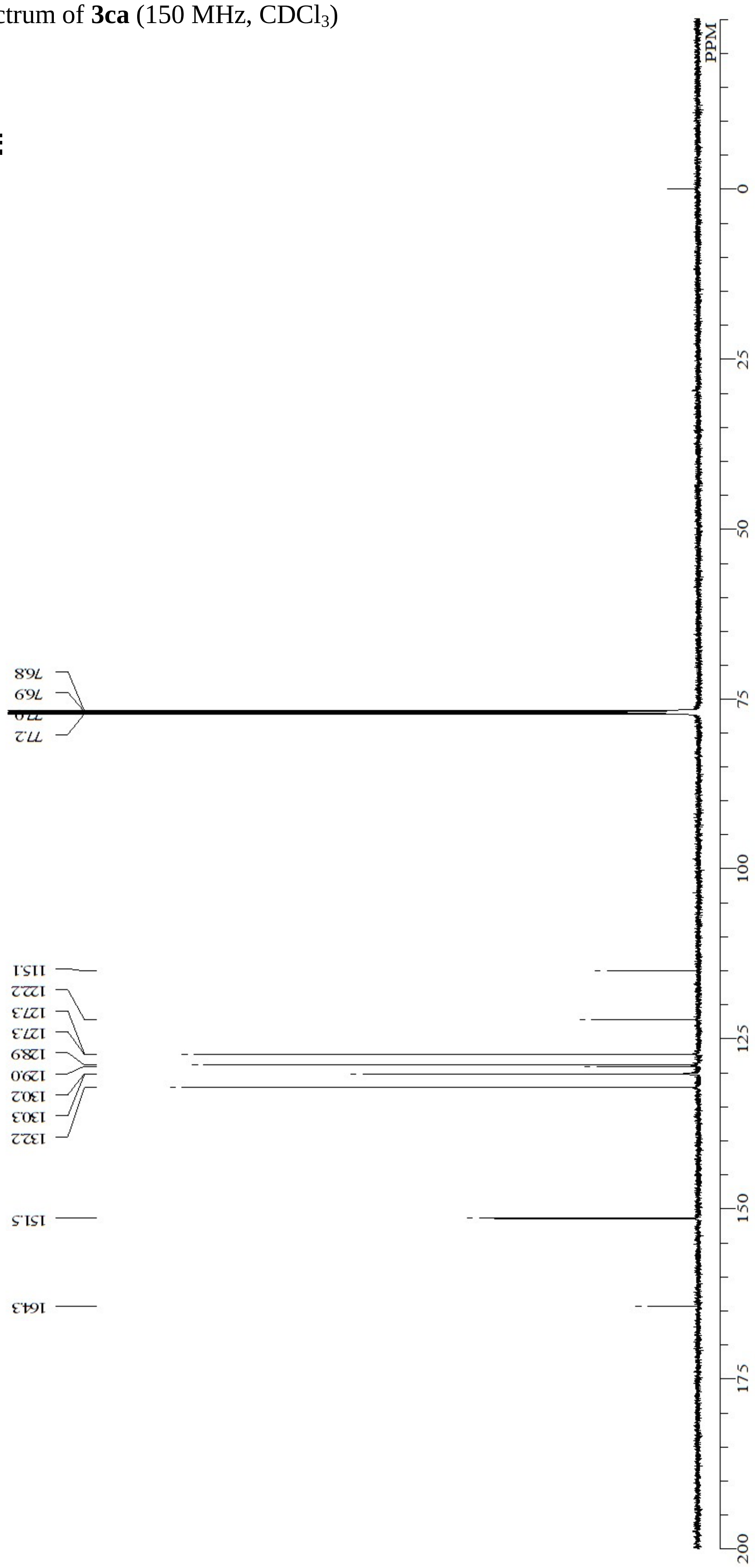

^{1}H NMR spectrum of **3da** (600 MHz, $CDCl_3$)

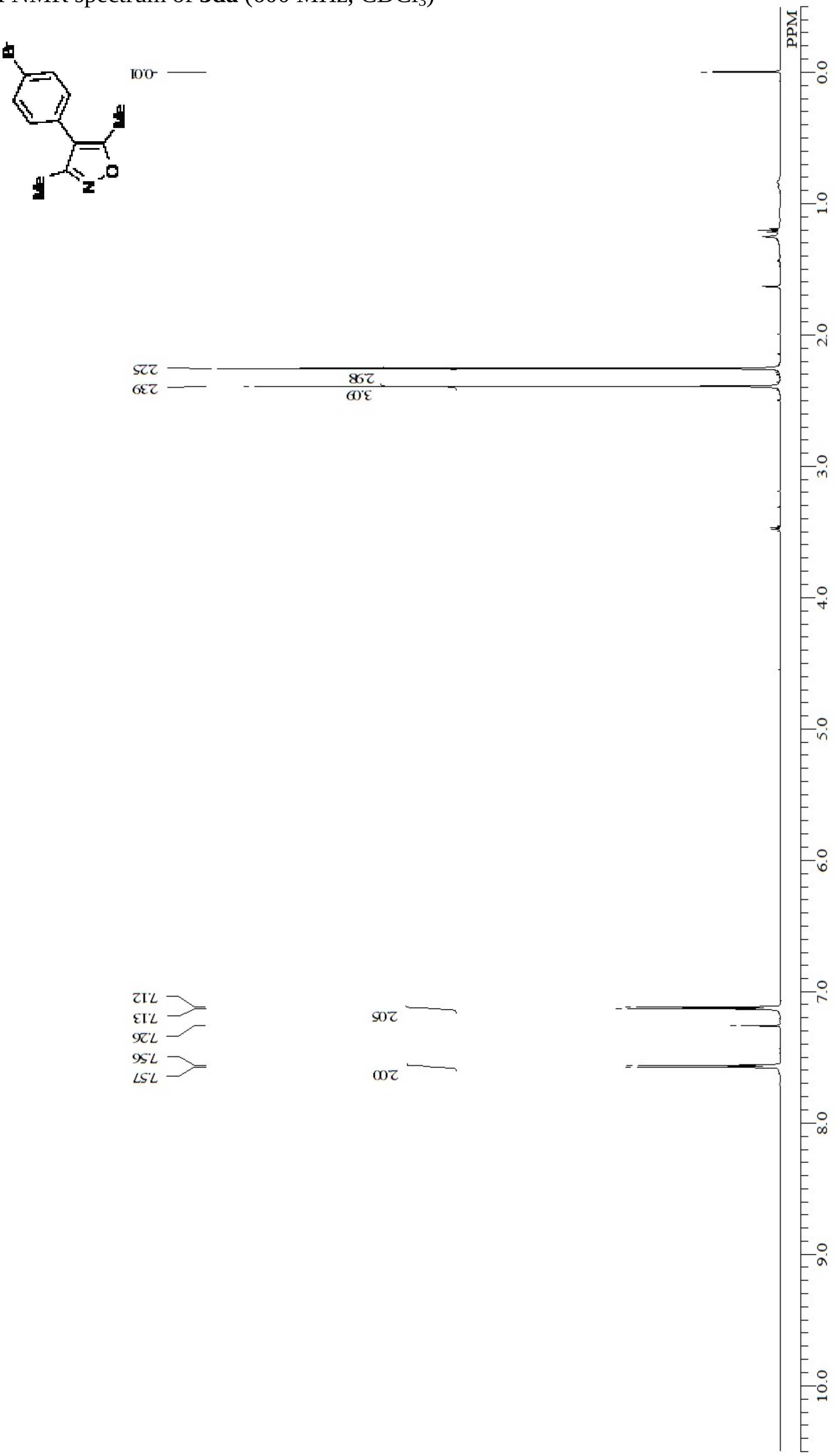

^{13}C NMR spectrum of **3da** (150 MHz, $CDCl_3$)

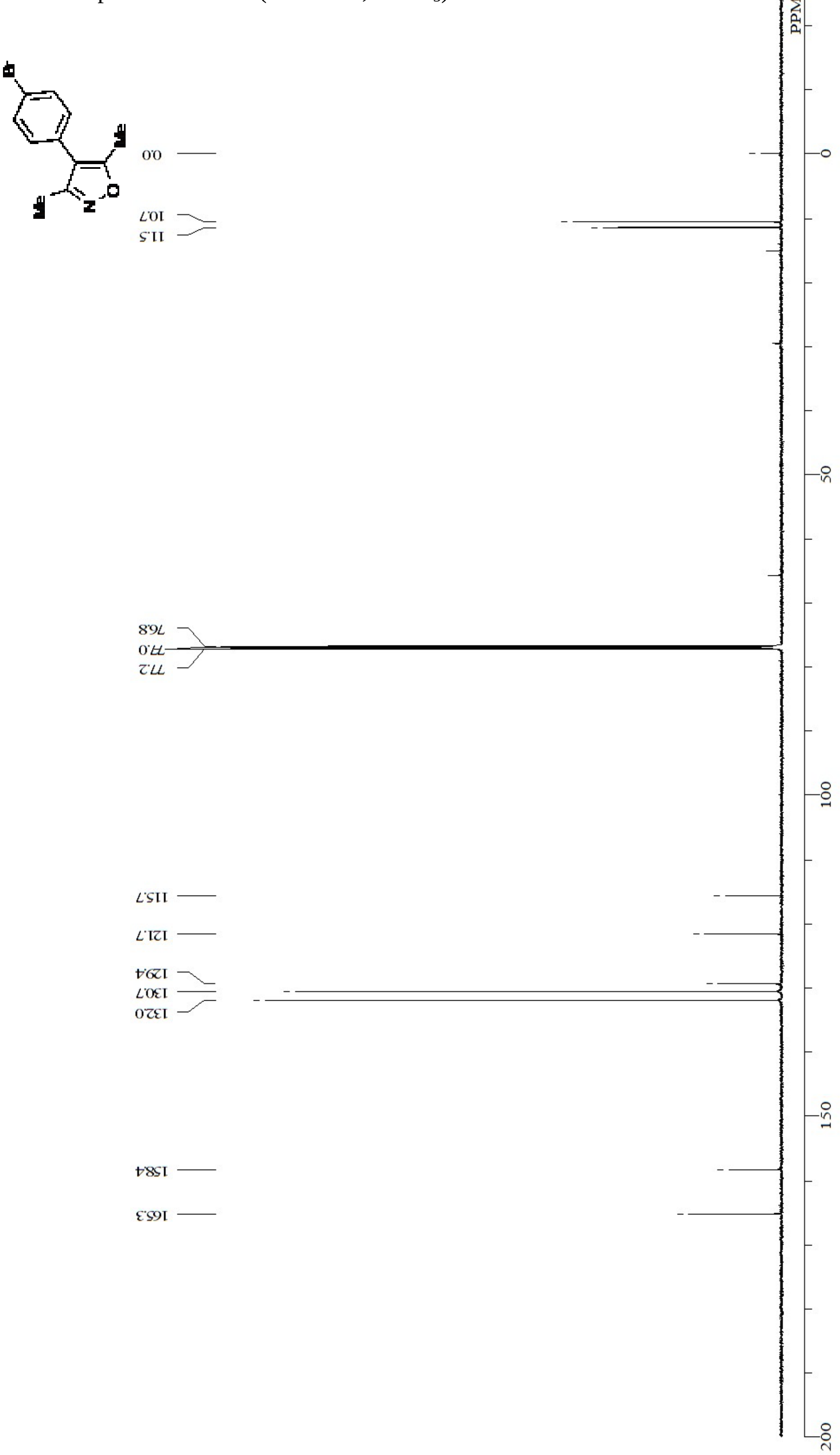

^{1}H NMR spectrum of **3ea** (600 MHz, $CDCl_3$)

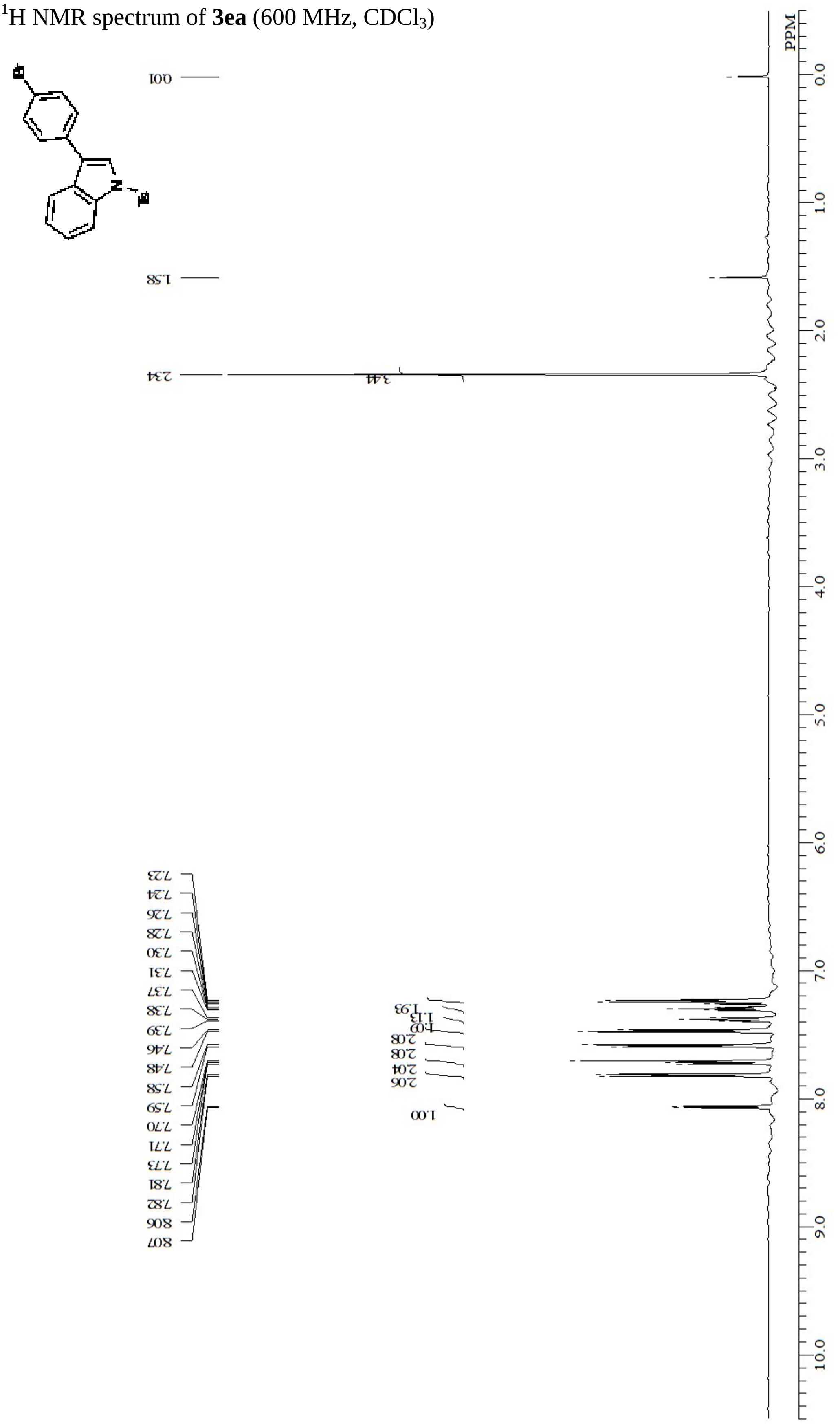

^{13}C NMR spectrum of **3ea** (150 MHz, $CDCl_3$)

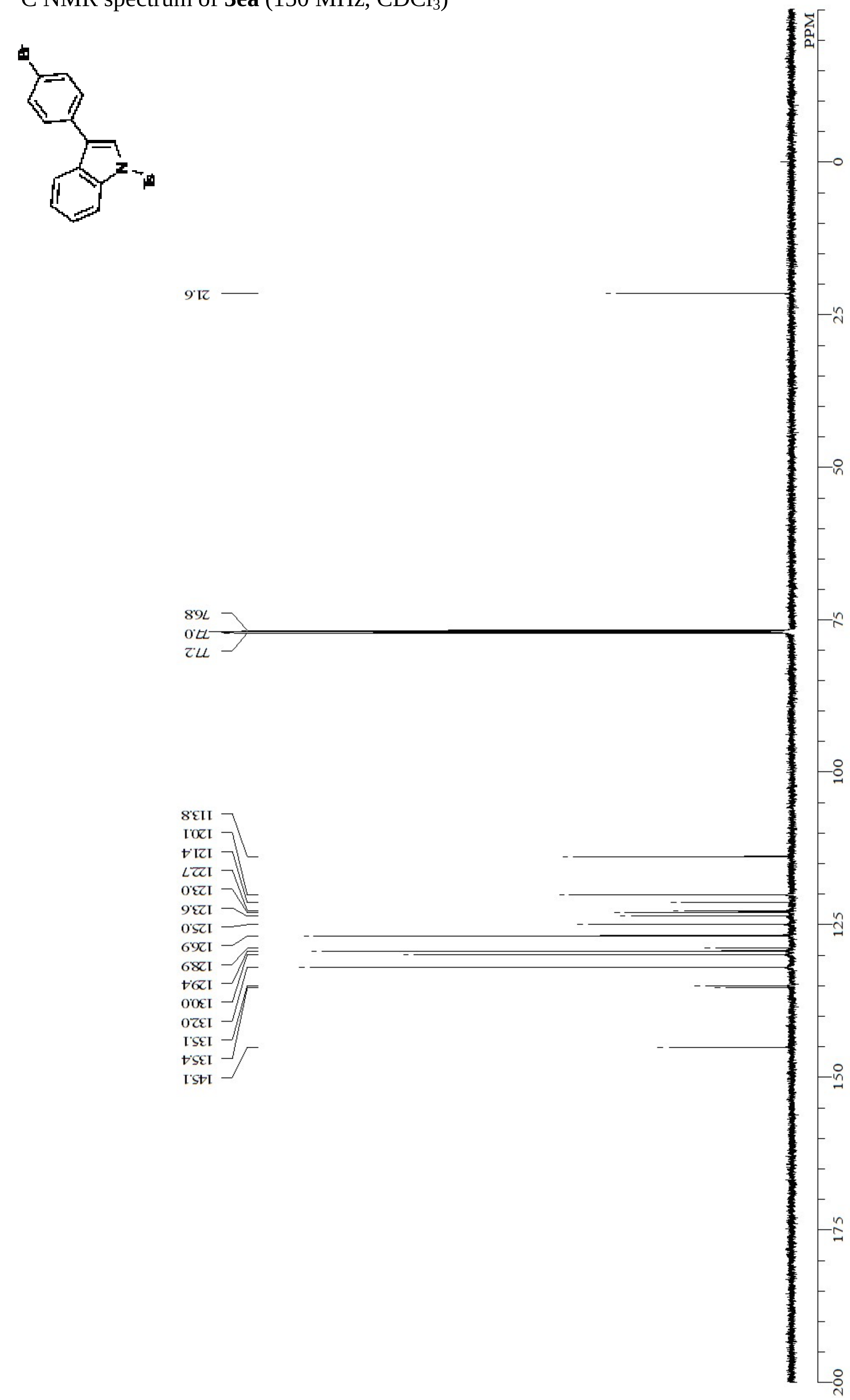

^{1}H NMR spectrum of **3fa** (600 MHz, $CDCl_3$)

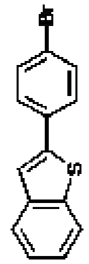

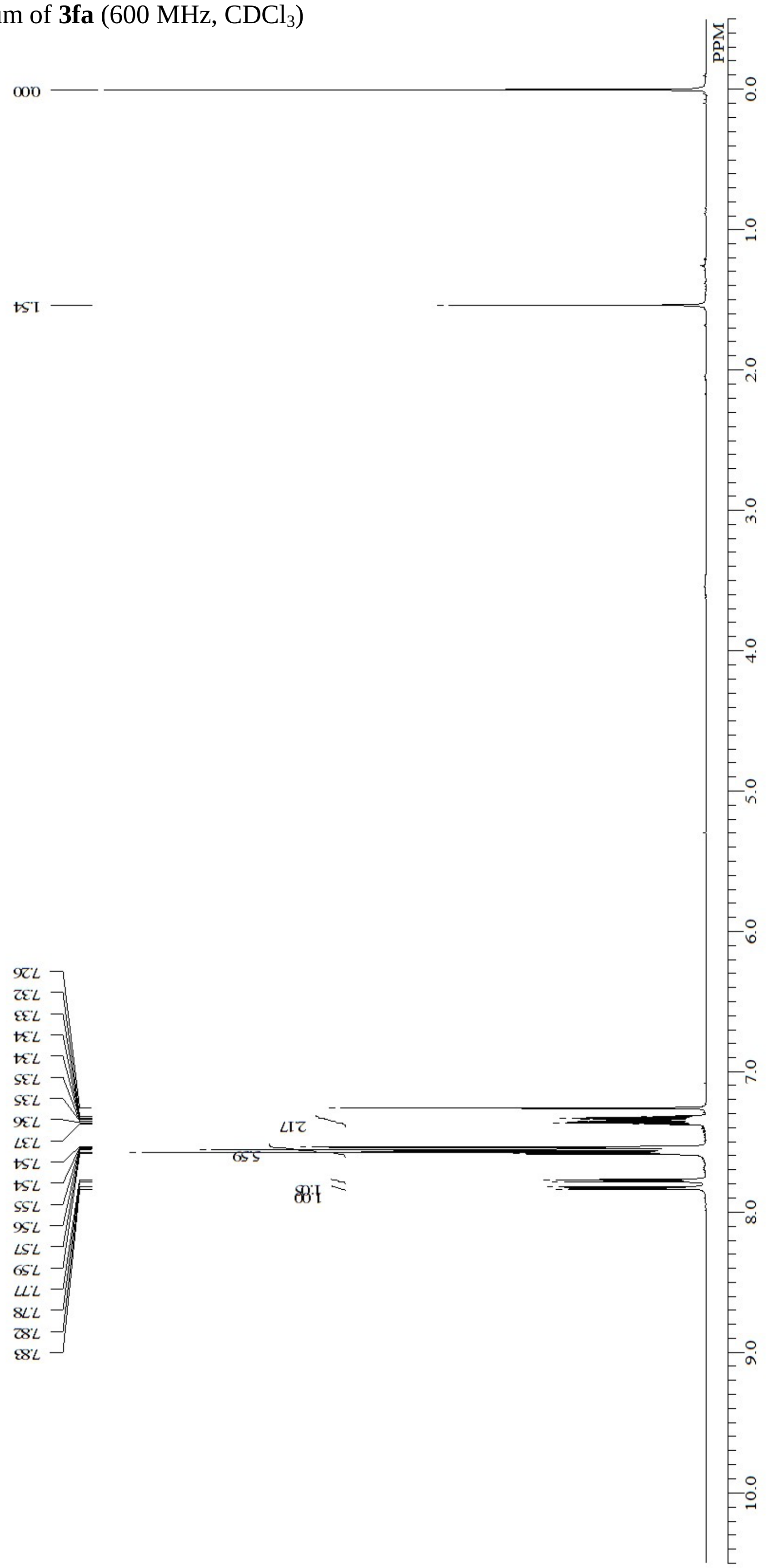

^{13}C NMR spectrum of **3fa** (150 MHz, $CDCl_3$)

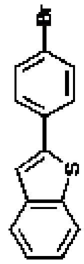

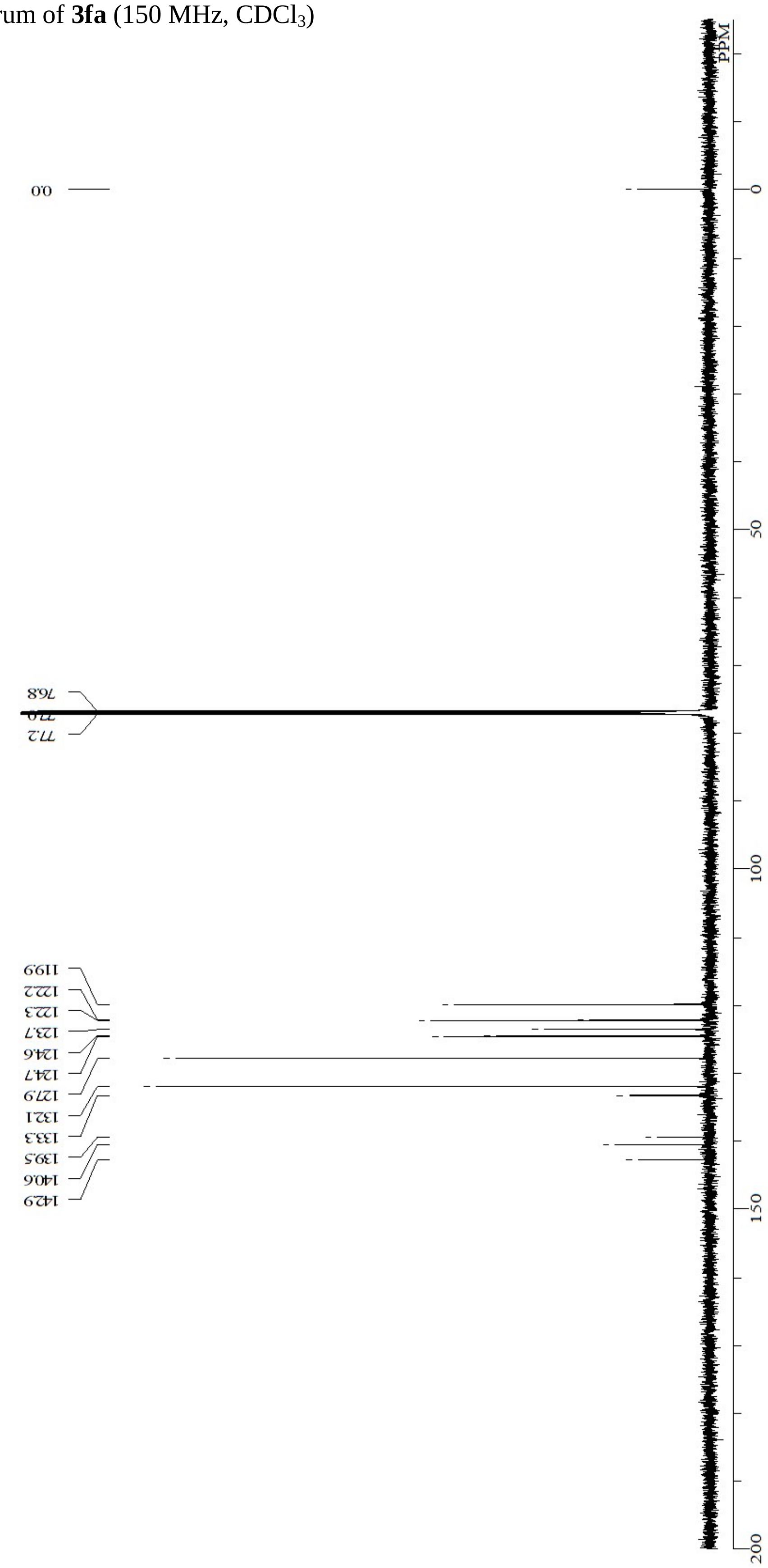

Supplement: File 1 — Experimental procedures, spectra of new compounds, CIF data, and details of the computational study. [file Beilstein_J_Org_Chem-11-2737-s001.pdf]
